# The m7G Methyltransferase Mettl1 Drives Cardiac Hypertrophy by Regulating SRSF9‐Mediated Splicing of NFATc4

**DOI:** 10.1002/advs.202308769

**Published:** 2024-05-29

**Authors:** Shuting Yu, ZhiYong Sun, Tiantian Ju, Yingqi Liu, Zhongting Mei, Changhao Wang, Zhezhe Qu, Na Li, Fan Wu, KuiWu Liu, Meixi Lu, Min Huang, Xiaochen Pang, Yingqiong Jia, Ying Li, Yaozhi Zhang, Shunkang Dou, Jianhao Jiang, Xianhui Dong, Chuanhao Huang, Wanhong Li, Yi zhang, Ye Yuan, Baofeng Yang, Weijie Du

**Affiliations:** ^1^ State Key Laboratory of Frigid Zone Cardiovascular Diseases (SKLFZCD) Department of Pharmacology (The State‐Province Key Laboratories of Biomedicine‐Pharmaceutics of China Key Laboratory of Cardiovascular Research Ministry of Education) College of Pharmacy Harbin Medical University Harbin 150081 China; ^2^ Traditional Chinese Medicine School Beijing University of Chinese Medicine Beijing 100013 China; ^3^ Department of Pharmacy (The University Key Laboratory of Drug Research Heilongjiang Province) The Second Affiliated Hospital of Harbin Medical University Harbin 150086 China; ^4^ Northern Translational Medicine Research and Cooperation Center Heilongjiang Academy of Medical Sciences Harbin Medical University Harbin 150081 China; ^5^ Research Unit of Noninfectious Chronic Diseases in Frigid Zone Chinese Academy of Medical Sciences 2019RU070 Harbin 150081 China

**Keywords:** alternative splicing, cardiac hypertrophy, m7G, Mettl1, NFATc4

## Abstract

Cardiac hypertrophy is a key factor driving heart failure (HF), yet its pathogenesis remains incompletely elucidated. Mettl1‐catalyzed RNA N7‐methylguanosine (m7G) modification has been implicated in ischemic cardiac injury and fibrosis. This study aims to elucidate the role of Mettl1 and the mechanism underlying non‐ischemic cardiac hypertrophy and HF. It is found that Mettl1 is upregulated in human failing hearts and hypertrophic murine hearts following transverse aortic constriction (TAC) and Angiotensin II (Ang II) infusion. YY1 acts as a transcriptional factor for Mettl1 during cardiac hypertrophy. Mettl1 knockout alleviates cardiac hypertrophy and dysfunction upon pressure overload from TAC or Ang II stimulation. Conversely, cardiac‐specific overexpression of Mettl1 results in cardiac remodeling. Mechanically, Mettl1 increases SRSF9 expression by inducing m7G modification of SRSF9 mRNA, facilitating alternative splicing and stabilization of NFATc4, thereby promoting cardiac hypertrophy. Moreover, the knockdown of SRSF9 protects against TAC‐ or Mettl1‐induced cardiac hypertrophic phenotypes in vivo and in vitro. The study identifies Mettl1 as a crucial regulator of cardiac hypertrophy, providing a novel therapeutic target for HF.

## Graphical abstract

This study elucidates a previously unknown role and the molecular mechanism by which Mettl1‐mediated m7G modification contributes to cardiac hypertrophy. The findings indicate that Mettl1 enhances the stability of SRSF9 through m7G modification, resulting in the upregulation of NFACT4 expression and the promotion of cardiac hypertrophy and heart failure progression. The graphical abstract is created using BioRender.

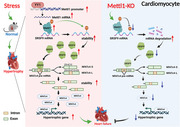

## 1 Introduction

Cardiac hypertrophy initially arises as a compensatory response of the myocardium to mechanistic stress provoked by various cardiac disorders. However, pathological cardiac hypertrophy, stemming from persistent stress, is characterized by structural and functional alterations in the heart and stands as an independent risk factor for the development of heart failure (HF).^[^ ^1^ ^]^ The pathogenesis of cardiac hypertrophy is profoundly intricate, involving neurohormone activation, disturbance of calcium handling, metabolic alterations, cell reprogramming, and the regulation of multiple signaling pathways.^[^ ^2^ ^]^ Despite significant progress in treating HF, the mechanisms underlying the transition from cardiac hypertrophy to HF largely remain elusive.

RNA epigenetic regulation, notably mediated by m6A, has been implicated in cardiac diseases, encompassing cardiac injury, fibrosis, and hypertrophy.^[^ ^3^, ^4^, ^5^, ^6^ ^]^ This highlights its potential as a novel therapeutic target for cardiac ailments. Another emerging RNA modification, N7‐methylguanosine (m7G), has been recently identified and is typically found in 5′ caps of mRNA,^[^ ^7^ ^]^ tRNAs,^[^ ^8^ ^]^ rRNAs,^[^ ^9^ ^]^ and mRNA.^[^ ^10^ ^]^ The m7G modification in RNA is catalyzed by the methyltransferase complex, comprising Mettl1 and WDR4.^[^ ^11^ ^]^ Studies have shown the involvement of Mettl1‐mediated m7G modification in the progression of various cancers as an oncogenic factor.^[^ ^12^, ^13^ ^]^ Additionally, Mettl1 has been linked to enhanced protein synthesis and angiogenesis after ischemia.^[^ ^14^, ^15^ ^]^ Recent research has shed light on the pathophysiological roles of Mettl1 in cardiomyocytes and fibroblasts in response to ischemic injury.^[^ ^16^, ^17^ ^]^ TMEM11 interacts with Mettl1 to facilitate m7G modification of ATF5 mRNA, thereby regulating cardiomyocytes proliferation. Moreover, fibroblast‐specific deficiency of Mettl1 has been shown to attenuate cardiac fibroblast activation and fibrosis following myocardial infarction.^[^ ^17^ ^]^ However, the role of Mettl1/m7G in non‐ischemic cardiac hypertrophy and HF remains unexplored.

In this study, we aimed to investigate the role and mechanism of Mettl1‐mediated m7G RNA modification in cardiac hypertrophy. Our study uncovers a previously unrecognized role of Mettl1 as a central driver of cardiac hypertrophy and remodeling under pressure overload, as demonstrated through using gain‐and‐loss‐of‐function studies conducted in vivo and in vitro. Furthermore, we elucidate that Mettl1 methylates SRSF9 mRNA to enhance its stability in an m7G‐dependent manner, consequently promoting alternative splicing and stabilization of NFATc4, thereby facilitating cardiac hypertrophic growth.

## 2 Results

### 2.1 Upregulation of Mettl1 Induced by YY1 During Cardiac Hypertrophy

**Figure 1:**
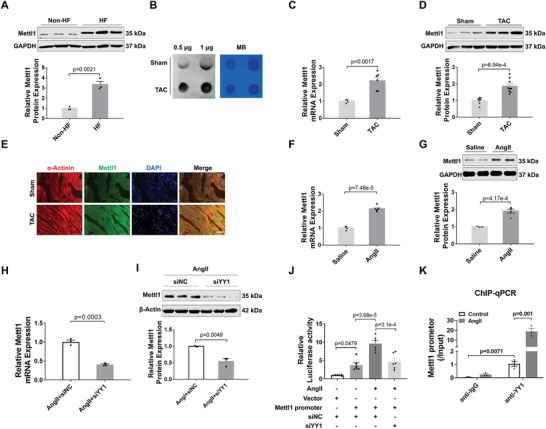
YY1 transcriptionally activates Mettl1 in hypertrophic mouse hearts. A) Western blotting analyses and quantification of Mettl1 protein levels in human heart tissues from heart failure patients and non‐heart failure patients (n = 3). B) Dot blot analysis of m7G modification levels in 10‐week TAC heart tissue, with methylene blue (MB) staining as control (n = 3). C) qRT‐PCR analysis of Mettl1 mRNA levels in the control group or the TAC group (n = 4–6). D) Western blot analysis of Mettl1 protein levels in 10‐week TAC heart tissue, with GAPDH as a control (n = 8‐9). E) Representative immunofluorescence images of α‐actinin‐ and Mettl1‐staining in the myocardial sections from the TAC‐ and sham‐operated groups, Scale bar: 50 µm (n = 3 mice). F) qRT‐PCR analysis of Mettl1 mRNA levels in heart tissue 4 weeks after Ang II infusion (n = 4‐5). G) Western blot analysis of Mettl1 protein levels in heart tissue 4 weeks after Ang II infusion (n = 5). H) qRT‐PCR was conducted to detect Mettl1 mRNA expression in NMCMs infected with YY1‐siRNA or YY1‐siNC followed by stimulation with Ang II (1 µm) for 48 h (n = 3). I) Protein levels of Mettl1 in NMCMs infected with YY1‐siRNA or YY1‐siNC followed by stimulation with Ang II (1 µm) for 48 h (n  = 3). J) NMCMs were infected with YY1‐siRNA or YY1‐siNC, an empty vector, or Mettl1 promoter followed by treatment with Ang II (1 µm) for 48 h. Luciferase activity was determined (n = 6). K) ChIP‐qPCR analysis was performed with YY1 or IgG antibody to determine the binding ability of YY1 to Mettl1 promoter in NMCMs after incubating with Ang II (1 µm) for 48 h (n = 3).

To investigate whether m7G methyltransferase Mettl1 is involved in cardiac hypertrophy, we assessed Mettl1 expression in the hearts of patients diagnosed with heart failure. Immunoblot assays revealed a significant increase in Mettl1 protein expression in failing hearts compared with non‐failing controls (**Figure** **1**A). To validate this finding, we established a mouse model of cardiac pressure overload induced by transverse aortic constriction (TAC). As depicted in Figure 1B and Figure S1A,B (Supporting Information), cardiac hypertrophic gene expression was upregulated in hypertrophic hearts, along with an increased level of m7G modification in total RNA. Western blot and qRT‐PCR analyses demonstrated a dramatic upregulation of Mettl1 mRNA and protein levels in hypertrophic hearts relative to sham‐operated controls (Figure 1C,D). Similarly, both mRNA and protein expression of Mettl1 were elevated in Ang II‐treated mouse hearts compared to the saline‐treated control group (Figure 1F,G). Moreover, immunofluorescent staining revealed that the increased cardiac protein expression of Mettl1 upon TAC was localized in cardiomyocytes, as evidenced by colocalization of Mettl1 with α‐actinin (Figure 1E). In line with the in vivo results, an Ang II‐induced cardiomyocyte hypertrophic model displayed remarkable increases in RNA m7G level and Mettl1 expression in neonatal mouse cardiomyocytes (NMCMs) (Figure S2A–C, Supporting Information). These findings suggest that Mettl1 may be involved in cardiac hypertrophy by mediating m7G modification.

Subsequently, we aimed to elucidate the upstream mechanism of Mettl1 expression during cardiac pressure overload. A recent study identified trans‐acting transcription factor 1 (SP1) and Yin Yang‐1 (YY1) as potential transcriptional factors of Mettl1, considering their implication in cardiac hypertrophy.^[^ ^18^, ^19^, ^20^, ^21^ ^]^ To investigate this, we constructed siRNAs for YY1 and SP1 and examined the alteration of Mettl1 expression after knocking down YY1 or SP1, respectively. In Ang II‐infused NMCMs, both mRNA and protein levels of Mettl1 were markedly reduced upon YY1 knockdown (Figure 1H,I, Figure S3A,B, Supporting Information). However, the knockdown of SP1 had no effect on Mettl1 expression (Figure S3C–F, Supporting Information). These results suggest that YY1 may act as a transcriptional factor of Mettl1. To confirm this hypothesis, we constructed luciferase vectors containing the Mettl1 promoter and transfected them into NMCMs treated with Ang II. The luciferase assay results showed that Ang II increased Mettl1 promoter activity, which was dramatically abrogated upon silencing YY1 (Figure 1J). Furthermore, chromatin immunoprecipitation followed by qPCR (ChIP‐qPCR) demonstrated YY1 binding to the Mettl1 promoter, with enhanced interaction upon Ang II treatment (Figure 1K). Together, these findings indicate that YY1 acts as an upstream regulator to activate Mettl1 expression during cardiac hypertrophy.

### 2.2 Deficiency of Mettl1 Attenuates Cardiac Hypertrophy and HF Induced by Cardiac Pressure Overload

**Figure 2:**
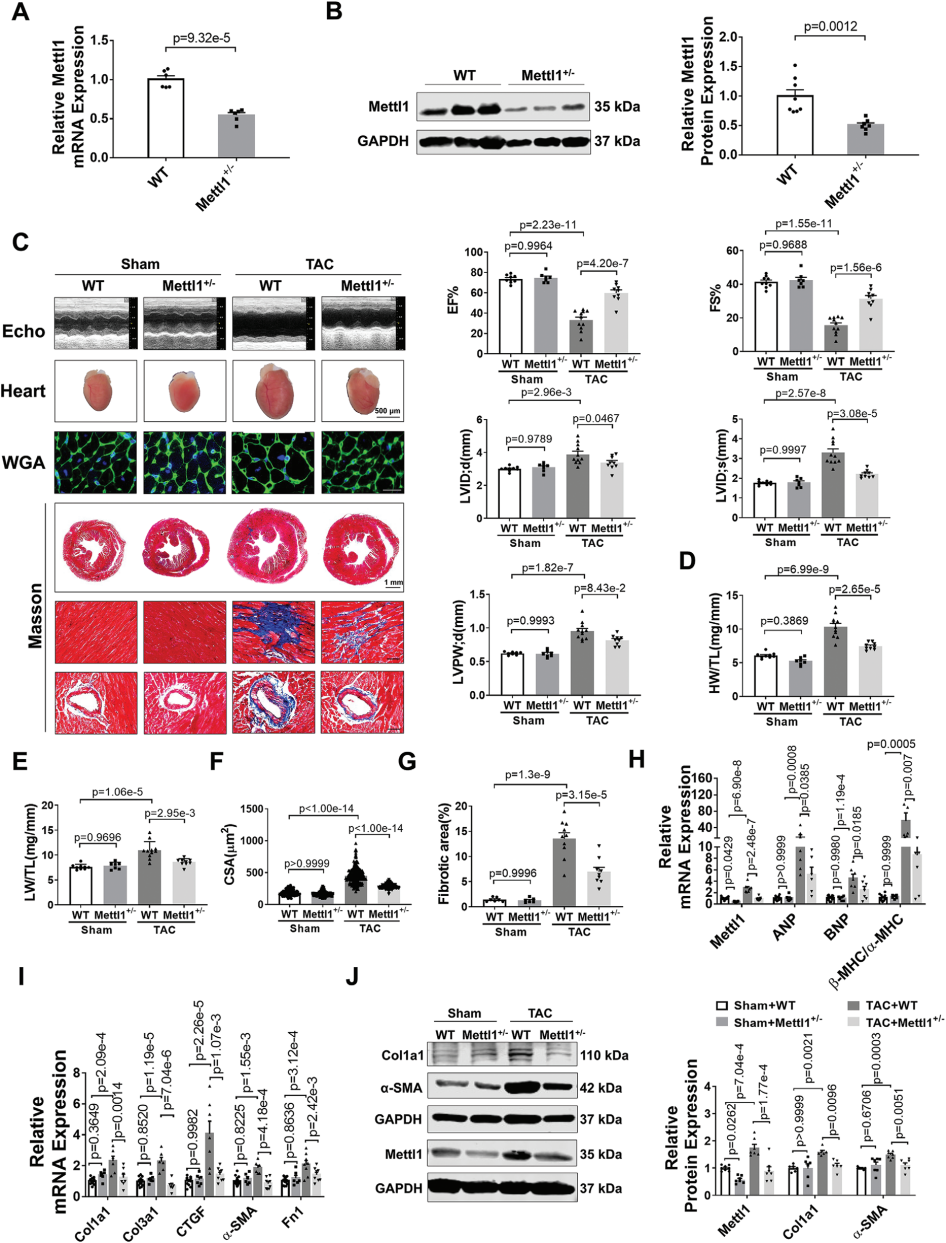
Deficiency of Mettl1 attenuates cardiac hypertrophy and HF following cardiac pressure overload. A) qRT‐PCR analysis of Mettl1 mRNA expression in 8–10‐week‐old WT and Mettl1^+/‐^ heart tissues (n = 6). B) Western blot analysis of Mettl1 protein levels in 8–10‐week‐old WT and Mettl1^+/‐^ heart tissues (WT: n = 8; Mettl1^+/−^: n = 7). C) Original traces of transthoracic M‐mode echocardiographic measurements from WT or Mettl1^+/−^ with 10‐week Sham and TAC mice. Echocardiographic parameters: EF%, the ejection fraction; FS%, the fraction shortening; LVID;d or LVID;s, the internal dimension of the left ventricle (LV), diastolic or systolic; and LVPW;d, the post wall thickness of LV (Sham+WT: n = 9; Sham+Mettl1^+/−^: n = 7; TAC+WT: n = 11; TAC+Mettl1^+/−^: n = 9). D) Relative heart weight (HW) to the tibia length (TL) of WT or Mettl1^+/−^ mice with sham and TAC for 10 weeks (Sham+WT: n = 9; Sham+Mettl1^+/−^: n = 7; TAC+WT: n = 11; TAC+Mettl1^+/−^: n = 9). E) Relative lung weight (LW) to the tibia length (TL) of WT or Mettl1^+/−^ mice with sham and TAC for 10 weeks (Sham+WT: n = 9; Sham+Mettl1^+/−^: n = 7; TAC+WT: n = 11; TAC+Mettl1^+/−^: n = 9). F) Quantification of cross‐sectional area (CSA) of ventricular cardiomyocytes shown in (C). A minimum of 60 cells were measured from different visual fields of six samples per group. Scale bar: 20 µm. G) Masson staining of WT or Mettl1^+/−^ mice with sham and TAC for 10 weeks. The relative fibrotic areas in whole heart sections are shown in (C). Scale bar: 50 µm (Sham+WT: n = 7; Sham+Mettl1^+/−^: n = 6; TAC+WT: n = 10; TAC+Mettl1^+/−^: n = 9). H) qRT‐PCR analysis on changes in hypertrophic markes in cardiac tissues of WT and Mettl1^+/−^ mice at 10 weeks after Sham or TAC surgery (n ≥ 6). I) qRT‐PCR analysis on changes in fibrosis marker expression in cardiac tissues from sham or TAC‐10 weeks WT and Mettl1^+/−^ mice (n ≥ 6). J) Western blot analysis of Col1a1, α‐SMA, and Mettl1 protein levels in heart tissue from WT or Mettl1^+/−^ mice with sham and TAC for 10 weeks (n ≥ 6).

To investigate the functional role of Mettl1 in cardiac hypertrophy, we generated whole‐body Mettl1 knockout mice (Figure S4A,B, Supporting Information). While the Mettl1 heterozygous knockout (Mettl1 KO) were viable and fertile, a significant reduction in cardiac Mettl1 mRNA and protein levels was confirmed in Mettl1 KO mice compared to their wild‐type (WT) littermates (**Figure** **2**A,B). Mettl1 KO and age‐matched WT littermates underwent TAC surgery to induce cardiac pressure overload, resulting in comparable increases in blood velocity, confirming equivalent levels of cardiac pressure overload (Figure S4C, Supporting Information). No significant differences were observed between Mettl1 KO and WT mice in terms of body weight, heart weight, and cardiac function (Figure 2C; Table S2, Supporting Information). However, 10 weeks after TAC, Mettl1 KO mice exhibited decreased ratios of heart weight to body weight (HW/WB), heart weight to tibia length (HW/TL), and lung weight to tibia length (LW/TL) relative to WT mice (Figure 2D,E, Table S2, Supporting Information). Echocardiographic analyses revealed improved cardiac function in Mettl1 KO mice post‐TAC compared to WT mice, as evidenced by increased ejection fraction (EF%) and fractional shortening (FS%). Additionally, TAC‐induced increases in LV internal diameter in systole (LVID;d) and diastole (LVID;s) and LV posterior wall thickness in diastole (LVPW;d) observed in WT mice were attenuated in Mettl1 KO mice (Figure 2C). Wheat germ agglutinin (WGA) staining demonstrated significantly reduced cardiomyocyte cross‐sectional area (CSA) in Mettl1 KO mice compared to WT mice post‐TAC (Figure 2F). Meanwhile, mRNA expression of cardiac fetal genes ANP, BNP, and β‐MHC/α‐MHC were downregulated in Mettl1 KO hearts compared to WT hearts post‐TAC (Figure 2H). Moreover, Mettl1 KO hearts exhibited pronounced reductions in cardiac perivascular and interstitial fibrosis post‐TAC, along with reduced expression of fibrotic genes Col1a1, Col3a1, CTGF, α‐SMA, and fibronectin 1 (Fn1), and the protein levels of Col1a1, and α‐SMA (Figure 2G,I,J).

To further investigate the role of Mettl1 in cardiac hypertrophy, Mettl1 KO mice and WT counterparts were subjected to Ang II infusion (2.5 mg kg^−1^ per day) for 4 weeks. No significant differences were observed in body weight, blood pressure, or heart rate between Mettl1 KO and WT mice post‐Ang II treatment (Table S3 and Figure S5E, Supporting Information). Ang II‐induced robust increases in Mettl1 mRNA and protein expression, which was attenuated in the myocardium of Mettl1 KO mice (Figure S5A,B, Supporting Information). Echocardiography revealed amelioration of cardiac dysfunction in Mettl1 KO mice post‐Ang II, as evidenced by increased EF% and FS%, decreased LVID;s, and LVPW;d (Figure S5C and Table S3, Supporting Information). Furthermore, Mettl1 KO mice exhibited less cardiac hypertrophy and reduced heart size, HW/TL, LW/TL, HW/BW, and cardiomyocytes CSA post‐Ang II relative to WT mice (Figure S5D–H and Table S3, Supporting Information). Consistent with these findings, the expression of cardiac fetal genes was lower in Mettl1 KO mice compared to WT controls post‐Ang II (Figure S5I, Supporting Information). These results suggest the Mettl1 deficiency protects against cardiac remodeling and HF in response to pressure overload.

### 2.3 Mettl1 Drives Cardiac Remodeling

**Figure 3:**
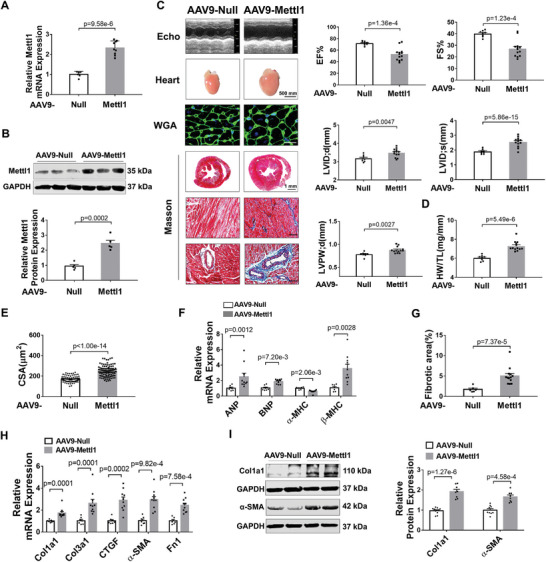
Mettl1 drives cardiac remodeling. A) qRT‐PCR analysis of Mettl1 mRNA expression in the myocardium from mice with AAV9‐Null or AAV9‐Mettl1 injection for 8 weeks (n = 6–9). B) Western blot analysis of Mettl1 protein levels in mouse hearts with AAV9‐Null or AAV9‐Mettl1 injection for 8 weeks (n = 5). C) The transthoracic M‐mode echocardiographic tracings from mice injected with AAV9‐Null or AAV9‐Mettl1 for 8 weeks. Echocardiographic parameters: EF%, FS%, LVID;d, LVID;s, and LVPW;d (AAV9‐Null: n = 8; AAV9‐Mettl1: n = 13). D) Relative HW/TL of mice injected with AAV9‐Null and AAV9‐Mettl1 (AAV9‐Null: n = 8; AAV9‐Mettl1: n = 13). E) Quantification of cross‐sectional area (CSA) of ventricular cardiomyocytes shown in (F). A minimum of 60 cells were measured from different visual fields of six samples per group. Scale bar: 20 µm. F) qRT‐PCR analysis for the mRNA levels of cardiac hypertrophic markers in AAV9‐Null or AAV9‐Mettl1‐treated mice (AAV9‐Null: n = 7; AAV9‐Mettl1: n = 10). G) Masson staining of AAV9‐Null or AAV9‐Mettl1‐treated mouse hearts. The relative fibrotic area in whole heart sections as shown in (C), Scale bar: 50 µm (AAV9‐Null: n = 6; AAV9‐Mettl1: n = 13). H) qRT‐PCR analysis on fibrotic markers in heart tissues from AAV9‐Null or AAV9‐Mettl1‐injected mice for 8 weeks (AAV9‐Null: n = 7; AAV9‐Mettl1: n = 10). I) Western blot analysis on Col1a1 and α‐SMA protein levels in heart tissues from mice injected with AAV9‐Null or AAV9‐Mettl1 for 8 weeks (n = 8–11).

The preceding results prompted further investigation into whether overexpression of Mettl1 could replicate the phenotypes observed in pressure overload‐induced cardiac remodeling. To address this question, we employed a gain‐of‐function strategy using adeno‐associated virus serotype 9 encoding Mettl1 transcript (AAV9‐Mettl1), with AAV9‐vector serving as the negative control (AAV9‐Null). Treatment with AAV9‐Mettl1 led to a significant upregulation of Mettl1 at both mRNA and protein levels compared to AAV9‐vector‐treated mice, reaching levels similar to those observed in TAC‐treated hearts (**Figures** **3**A,B and 1C,D). Notably, overexpression of Mettl1 markedly reduced EF% and FS% and caused cardiac dilatation (Figure 3C; Table S4, Supporting Information). Cardiac hypertrophy was also evident in Mettl1‐overexpressing mice, as evidenced by a marked increase in LVPW;d, HW/TL, and CSA (Figure 3C–E). Additionally, we assessed the surface area of adult mouse cardiomyocytes isolated from AAV9‐Null‐ and AAV9‐Mettl1‐treated mice. Consistently, the surface area of Mettl1‐overexpressing cardiomyocytes was increased compared to AAV9‐Null‐treated mice. Similarly, Mettl1 overexpression led to a pronounced increase in width and a moderate increase in the length of isolated cardiomyocytes (Figure S6A, Supporting Information). This was further supported by the marked elevation in mRNA expression of cardiac fetal genes following overexpression of Mettl1 (Figure 3F). Furthermore, Mettl1 overexpression induced cardiac fibrosis, as demonstrated by Masson's staining, along with a remarkable increase in mRNA expression of fibrotic genes, as well as increased protein levels of Col1a1 and α‐SMA (Figure 3G–I).

Next, we conducted an in vitro study to explore the role of Mettl1 in cardiomyocyte hypertrophy. To this end, siRNA targeting Mettl1 was transfected into NMCMs before treatment with Ang II. siMettl1 treatment resulted in a significant decrease in Mettl1 protein levels in NMCMs (Figure S7A, Supporting Information). Ang II‐induced cell enlargement, as detected by immunofluorescence staining, was prevented by the knockdown of Mettl1 (Figure S7B, Supporting Information). Similarly, the knockdown of Mettl1 attenuated Ang II‐induced upregulation of ANP, BNP, and β‐MHC mRNA expression (Figure S7C, Supporting Information). Conversely, artificial overexpression of Mettl1 by infecting NMCMs with adenovirus (Adv) containing the Mettl1 gene yielded opposite results (Figure S7D–F, Supporting Information). Specifically, Mettl1 overexpression led to cardiomyocyte hypertrophy (Figure S7E, Supporting Information), as indicated by increases in cardiomyocyte size and ANP, and BNP mRNA levels (Figure S7F, Supporting Information). These results collectively demonstrate that Mettl1 is sufficient to induce cardiac remodeling.

### 2.4 SRSF9 as a Target of Mettl1‐Mediated m7G Modification

**Figure 4:**
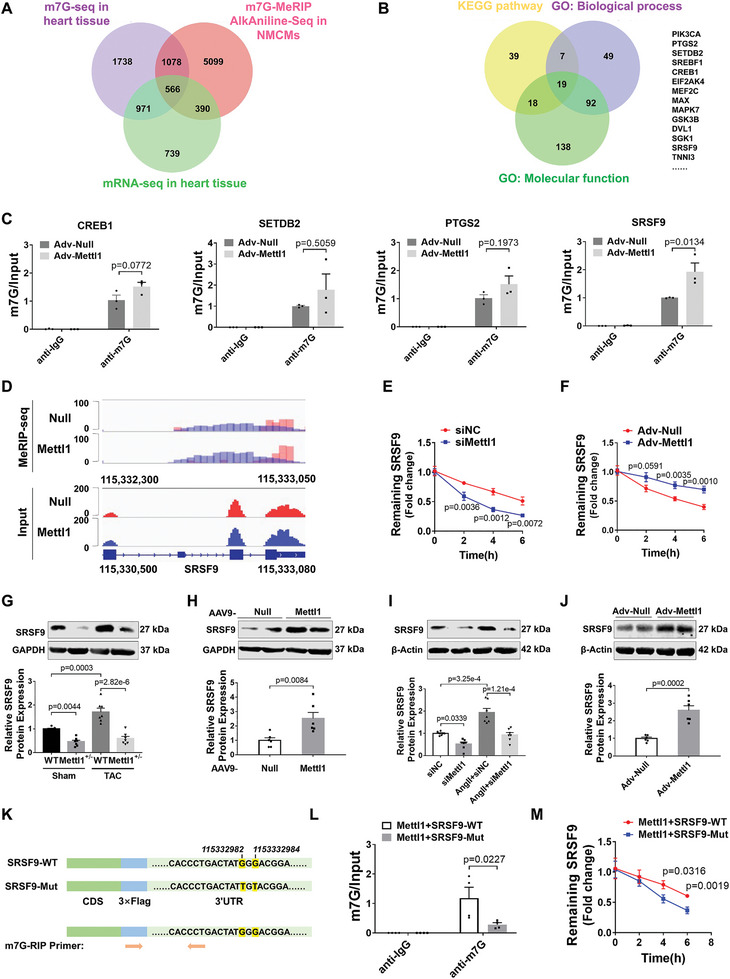
SRSF9 is a target of Mettl1‐mediated m7G modification. A) A cross‐tabulation analysis of MeRIP‐seq showing the intersections of upregulated genes (AAV9‐Null vs AAV9‐Mettl1, FC > 1.5) following enhanced m7G modifications in NMCMs (Adv‐Null vs Adv‐Mettl1, FC > 3) and myocardium (AAV9‐Null vs AAV9‐Mettl1, FC > 1.5). B) The Vann plot shows the 19 potential target genes of Mettl1 obtained from screening based on combined KEGG and GO analyses. C) Effects of Mettl1 on the m7G modification of CREB1, SETDB2, PTGS2, and SRSF9 assessed by RNA‐binding protein immunoprecipitation (RIP) in NMCM transfected with Adv‐null or Adv‐Mettl1 (n = 3). D) Visualization of the m7G methylation site on the SRSF9 gene. E) The decay rate of SRSF9 analyzed by qRT‐PCR in NMCMs treated with 5 µg mL^−1^ actinomycin D for 0, 2, 4, and 6 h after transfection with siNC or siMettl1 for 48 h (n = 7–9). F) The decay rate of SRSF9 determined by qRT‐PCR in NMCMs treated with 5 µg mL^−1^ actinomycin D for 0, 2, 4, and 6 h after transfection with Adv‐Null or Adv‐Mettl1 for 48 h (n = 8–9). G) Western blot analysis of SRSF9 protein levels in heart tissues from WT or Mettl1^+/−^ mice of the sham and TAC groups (n = 7). H) Western blot analysis of SRSF9 protein levels in cardiac tissues from mice treated with AAV9‐Null or AAV9‐Mettl1 for 8 weeks (n = 6). I) Western blot analysis of SRSF9 protein levels in NMCMs transfected with siNC or siMettl1 and pretreated with Ang II for 48 h (n = 7). J) Western blot analysis of SRSF9 protein levels in NMCMs treated with Adv‐Null or Adv‐Mettl1 for 48 h (n = 6). K) A graphical illustration of the construction of the plasmid carrying SRSF9 mutation sites (G‐to‐T mutation) and the specific primer was designed for MeRIP‐qPCR and RNA decay analysis. L) SRSF9 wild‐type (SRSF9‐WT, CDS+3′UTR) or SRSF9‐mutant (SRSF9‐Mut, CDS+3′UTR‐mutant) plasmids were co‐transfected with Adv‐Mettl1 into NMCMs, respectively. The effect of Mettl1 on m7G modification of exogenous SRSF9 transcript was assessed by RIP‐qPCR with specific primer as indicated in Figure 4K (n = 4). M) The decay rates of exogenous SRSF9 transcript analyzed by qRT‐PCR in NMCM treated with 5 µg mL^−1^ actinomycin D for 0, 2, 4, and 6 h after transfection with Adv‐Mettl1 for 48 h (n = 6).

To elucidate the mechanism by which Mettl1 drives cardiac hypertrophy, we employed RNA‐seq combined with m7G methylated RNA sequencing (m7G‐seq) for whole transcriptome profiling and m7G sites mapping in Mettl1‐overexpressing mouse myocardium and NMCMs, respectively. Total m7G methylation levels were higher in NMCMs after Mettl1 overexpression, whereas Mettl1 knockdown had the opposite effect (Figure S8A, Supporting Information). The m7G‐seq analysis revealed 3085 upregulated genes with 5372 hypermethylated peaks in Mettl1‐overexpressing hearts (Figure S8B,C, Supporting Information). Furthermore, the correlation between the levels of m7G peaks and gene expression demonstrated an increase in m7G modifications in 1520 up‐regulated genes (Figure S8D, Supporting Information). After intersecting the m7G‐seq and m7G AlkAniline‐Seq results, we identified 566 overlapped genes with increased m7G peaks and mRNA expression after Mettl1 overexpression (**Figure** **4**A). Kyoto Encyclopedia of Genes and Genomes (KEGG) analysis indicated that the 566 overlapping genes were mainly enriched in signaling pathways associated with cardiac hypertrophy, such as AMPK, MAPK, and mTOR pathways (Figure S8E, Supporting Information). Gene Ontology (GO) analysis on the 566 overlapping genes revealed participation in biological processes is relevant to cardiac development, angiogenesis, and DNA damage (Figure S8F, Supporting Information). Moreover, several genes exhibited protein and nucleic acid binding capability (Figure S8G, Supporting Information). To identify the specific target of Mettl1, we cross‐referenced the results of KEGG and GO analyses, resulting in 14 probable genes that may be involved in cardiac hypertrophy‐related pathways (Figure 4B). We then assessed the expression of these candidate genes in TAC‐treated and Mettl1‐overexpressing hearts, revealing that 4 out of 14 genes exhibited consistent upregulation in both conditions (Figure S9A–C, Supporting Information). Furthermore, m7G immunoprecipitation‐qPCR results demonstrated that m7G methylated SRSF9 mRNA was significantly immunoprecipitated by anti‐m7G compared to anti‐IgG, and this enrichment was remarkably enhanced by Mettl1 overexpression in NMCMs and mouse myocardium (Figure 4C; Figure S9D, Supporting Information). Conversely, TAC‐induced elevation of m7G level of SRSF9 mRNA was attenuated in Mettl1‐deficient hearts (Figure S9D, Supporting Information). Integrative Genomics Viewer analysis displayed markedly increased m7G levels and mRNA expression of SRSF9 in NMCMs after Mettl1 overexpression (Figure 4D). To assess whether Mettl1 affects the stability of SRSF9 mRNA, we performed RNA decay assays in NMCMs. The results revealed that knockdown of Mettl1 accelerated the decay of SRSF9 mRNA, whereas overexpression of Mettl1 prevented SRSF9 mRNA decay in cardiomyocytes after actinomycin D treatment (Figure 4E,F). Mettl1 KO hearts exhibited a marked decrease in SRSF9 protein expression in the presence and absence of TAC surgery (Figure 4G). Conversely, Mettl1 overexpression significantly elevated SRSF9 protein levels (Figure 4H). Similar results were observed in NMCMs in vitro (Figure 4I,J).

The m7G AlkAniline‐Seq analysis indicated that two m7G peaks were located at the 3′UTR of SRSF9 mRNA. Moreover, we predicted the binding preference of Mettl1 to SRSF9 mRNA via the catRAPID database, and the results showed that Mettl1 has a stronger binding preference for the 3′UTR region of SRSF9 mRNA (Figure S9E, Supporting Information). To further confirm that SRSF9 was a direct target of Mettl1, we constructed a plasmid containing the wild‐type SRSF9 coding sequences (CDS) and 3′UTR regions (SRSF9‐WT), and a mutant SRSF9 3′UTR (SRSF9‐Mut) plasmid (Figure 4K). Western blotting verified that both plasmids significantly overexpressed SRSF9 in NMCMs (Figure S9F, Supporting Information). As anticipated, m7G RIP‐qPCR results showed that SRSF9‐WT, but not SRSF9‐Mut, was able to be immunoprecipitated by anti‐m7G (Figure 4L). Additionally, Mettl1 facilitated the stability of transfected exogenic SRSF9 transcript in SRSF9‐WT‐treated cardiomyocytes, whereas this effect was abolished in SRSF9‐Mut‐treated cardiomyocytes (Figure 4M). These results demonstrate that Mettl1 regulates SRSF9 expression in an m7G‐dependent manner.

### 2.5 Deficiency of SRSF9 Dampens Cardiac Remodeling

**Figure 5:**
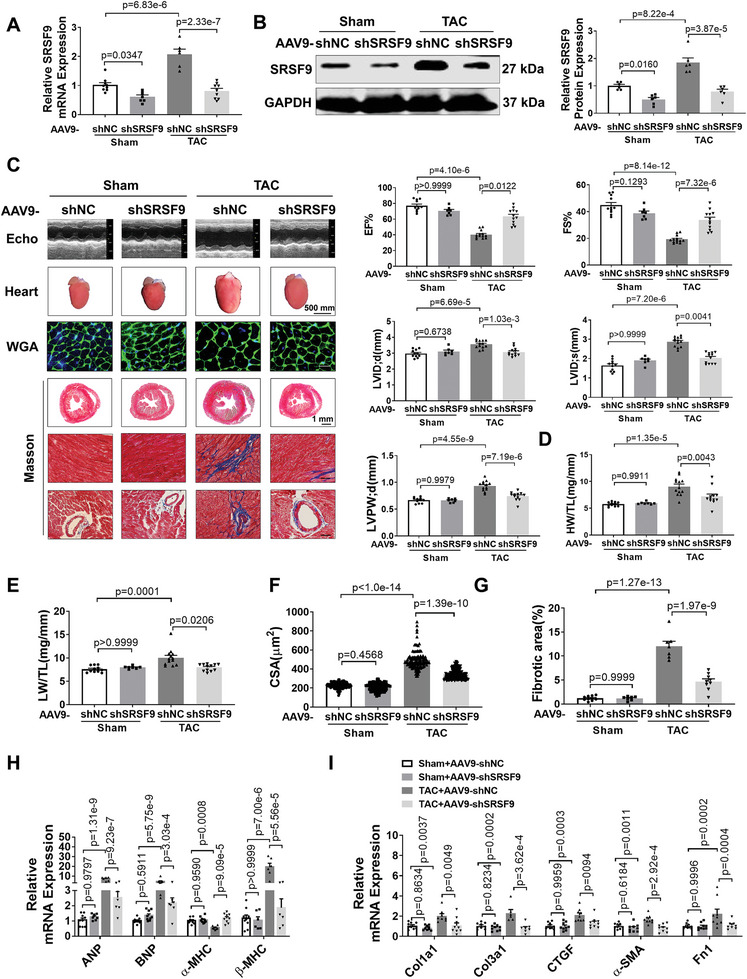
Deficiency of SRSF9 abrogates cardiac remodeling. A) qRT‐PCR quantification of changes in SRSF9 transcripts in heart tissues treated with AAV9‐shNC or AAV9‐shSRSF9 in the sham and TAC groups, respectively (n = 6–9). B) Western blot analysis of SRSF9 protein levels in heart tissues from 10‐week sham or TAC mice treated AAV9‐shNC or AAV9‐shSRSF9 (n = 6). C) The original traces of transthoracic M‐mode echocardiographic recordings from 10‐week Sham and TAC mice treated with AAV9‐shNC or AAV9‐shSRSF9. Echocardiographic parameters: EF%, FS%, LVID;d or LVID;s, LVPW;d (Sham+AAV9‐shNC: n = 11; Sham+AAV9‐shSRSF9: n = 7; TAC+AAV9‐ shNC: n = 13; TAC+AAV9‐shSRSF9: n = 12). D) Relative heart weight (HW) to the tibia length (TL) in 10‐week sham and TAC mice transfected with AAV9‐shNC or AAV9‐shSRSF9 (Sham+AAV9‐ shNC: n = 11; Sham+AAV9‐shSRSF9: n = 7; TAC+AAV9‐ shNC: n = 13; TAC+AAV9‐shSRSF9: n = 12). E) Relative lung weight (LW) to the tibia length (TL) in 10‐week sham and TAC mice transfected with AAV9‐shNC or AAV9‐shSRSF9 (Sham+AAV9‐ shNC: n = 11; Sham+AAV9‐shSRSF9: n = 7; TAC+AAV9‐ shNC: n = 13; TAC+AAV9‐shSRSF9: n = 12). F) Quantification of cross‐sectional areas (CSA) of ventricular cardiomyocytes shown in (C). A minimum of 60 cells were measured from different microscopic fields of six samples per group. Scale bar: 20 µm. G) Masson staining of the cardiac tissues from 10‐week sham and TAC mice treated with AAV9‐shNC or AAV9‐shSRSF9. Relative fibrotic areas in whole heart sections are shown in (C). Scale bar: 50 µm (Sham+AAV9‐ shNC: n = 10; Sham+AAV9‐shSRSF9: n = 7; TAC+AAV9‐ shNC: n = 7; TAC+AAV9‐shSRSF9: n = 9). H) qRT‐PCR analysis on the expression of cardiac hypertrophic marker genes in myocardial tissues from 10‐week sham or TAC mice infected with AAV9‐shNC or AAV9‐shSRSF9 (n = 7–9). I) qRT‐PCR analysis for fibrotic marker genes in myocardial tissues from 10‐week sham or TAC mice treated with AAV9‐shNC or AAV9‐shSRSF9 (n = 7–9).

To examine the function of SRSF9 in cardiomyocyte hypertrophy, we designed three different siRNA sequences to silence SRSF9 in NMCMs. The results showed that treatment with siSRSF9 2# produced the most remarkable downregulation of SRSF9 protein expression (Figure S10A, Supporting Information). Knockdown of SRSF9 attenuated Ang II‐induced increases in cardiomyocyte size and the expression of hypertrophic genes, while enforced overexpression of SRSF9 significantly increased cardiomyocyte hypertrophy (Figure S10B–H, Supporting Information). To corroborate our in vitro results, we performed an in vivo loss‐of‐function study to explore the role of SRSF9 in cardiac hypertrophy. To this end, we constructed an AAV9 carrying an SRSF9‐shRNA fragment (AAV9‐shSRSF9) to knock down SRSF9 in mouse myocardium. The mice were subjected to an equal level of TAC surgery, as demonstrated by comparable increases in aortic arch blood flow rate (Figure S11A, Supporting Information). Administration of AAV9‐shSRSF9 significantly reduced SRSF9 mRNA and protein levels and markedly abrogated TAC‐induced increase in SRSF9 expression relative to AAV9‐shNC‐treated mice (**Figure** **5**A,B). Echocardiographic analysis showed that the knockdown of SRSF9 significantly alleviated cardiac dysfunction, reduced cardiac dilatation, and LVPW;d induced by TAC (Figure 5C; Table S5, Supporting Information). Moreover, the knockdown of SRSF9 attenuated an increase in HW/TL and LW/TL (Figure 5D,E). WGA staining showed that TAC induced a marked increase in CSA, which was abrogated by silencing SRSF9 (Figure 5F). Consistently, TAC‐induced increased expression levels of cardiac hypertrophic genes were abrogated by knockdown of SRSF9 (Figure 5H). In addition, inhibition of SRSF9 decreased cardiac fibrosis and the expression of fibrotic genes after TAC (Figure 5G,I). These results suggest that the blockade of SRSF9 alleviates cardiac hypertrophy upon pressure overload.

To investigate whether overexpression of SRSF9 results in the same phenotype as pressure overload‐induced cardiac hypertrophy, a gain‐of‐function approach was employed by infecting with AAV9‐SRSF9 and its negative control AAV9‐Null. AAV9‐SRSF9 treatment resulted in a significant upregulation of SRSF9 at mRNA and protein levels as compared with AAV9‐vector‐treated mice (Figure S12A,B, Supporting Information). Notably, overexpression of SRSF9 markedly reduced EF% and FS% and caused cardiac dilatation (Figure S12C, Supporting Information). Cardiac hypertrophy was also observed in SRSF9‐overexpressing mice as displayed by a marked increase in LVPW;d, HW/TL, and CSA (Figure S12C–E, Supporting Information). This was further verified by the results that enforced expression of SRSF9 led to a pronounced increase in cardiac fetal gene expression (Figure S12F, Supporting Information).

### 2.6 SRSF9 Stabilizes NFATc4 mRNA by Regulating Splicing

**Figure 6:**
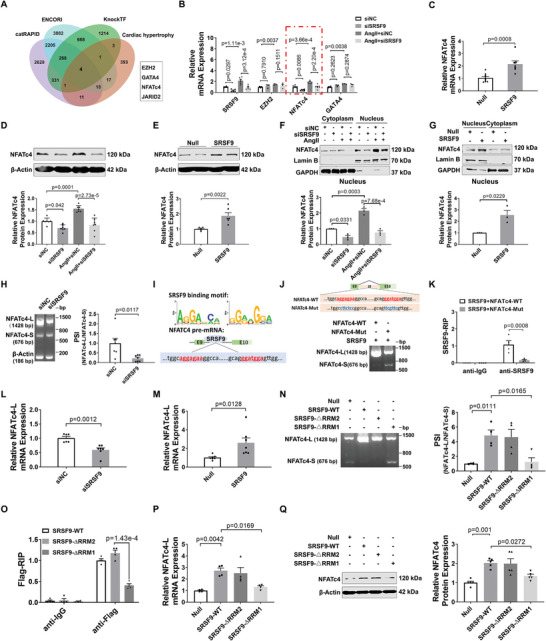
SRSF9 stabilizes NFACT4 mRNA by regulating splicing. A) The Venn diagram illustrates SRSF9's potential targets as predicted by three databases: catRAPID (http://service.tartaglialab.com/page/catrapid_group), ENCORI (https://rnasysu.com/encori/index.php), and KnockTF (http://www.licpathway.net/KnockTF/index.html). Identification of potential targets for SRSF9 and analysis of the association between cardiac hypertrophy‐regulated genes in GO annotation (https://www.ebi.ac.uk/QuickGO/, GO: 0 003300, GO:00 10611) and published research. B) qRT‐PCR analysis of EZH2, NFATc4, and GATA4 mRNA levels in NMCM transfected with siNC or siSRSF9 and pretreated with Ang II for 48 h (EZH2 and GATA4: n = 3, SRSF9 and NFATc4: n = 6). C) qRT‐PCR analysis of NFATc4 transcripts in NMCMs treated with SRSF9‐plasmid (1 µg mL^−1^) for 48 h (n = 7–9). D) Changes in protein levels of NFATc4 in the NMCMs transfected with siSRSF9 and pretreated with Ang II for 48 h (n = 7). E) Western blot analysis changes in NFATc4 protein levels in NMCMs transfected with SRSF9‐plasmid (1 µg mL^−1^) for 48 h (n = 6). F) SRSF9 was silenced and the cytoplasm and nucleus of NMCMs with or without Ang II induction were isolated for quantification of NFATc4 protein levels (n = 3). G) The cytoplasm and nucleus of NMCMs were isolated after SRSF9 overexpression. NFATc4 protein levels was determined by Western blotting (n = 3). H) Intron retention in NFATc4 was investigated in NMCMs with silencing of SRSF9. Agarose gel electrophoresis was performed with the percent spliced‐in (PSI) primers as shown in Figure S13D (Supporting Information). I) Top: The RBPmap database (http://rbpmap.technion.ac.il/) predicts binding motifs for SRSF9; Bottom: integrated genome viewer tracks centers around the NFATc4 gene. SRSF9 binding motifs are indicated in red. J) Top: The schematic of NFATc4 splicing reporters with the predicted SRSF9‐binding site in red and the mutated site in blue. Bottom: The SRSF9 plasmid co‐infected with NFATc4‐WT or NFATc4‐Mut for investigating in vitro splicing of NFATc4 minigene reporter transcripts in NMCMs. The PSI primers are depicted in Figure S13D (Supporting Information), with the bands indicating the size of the splicing event. (n = 3). K) Binding of NFATc4 pre‐mRNAs with SRSF9 was examined by RNA‐immunoprecipitation assay in NMCMs exogenously expressed SRSF9. Two pairs primers were used to examine NFATc4‐WT or NFATc4‐Mut, respectively as shown in Figure S13E (Supporting Information) (n = 4). L) The mRNA expression of NFATc4‐L was confirmed by qRT‐PCR in NMCMs transfected with siSRSF9 for 48 h (n = 6‐7). M) The mRNA expression of NFATc4‐L was confirmed by qRT‐PCR in NMCMs transfected with SRSF9‐plasmid for 48 h (n = 7). N) Intron retention in NFATc4 was investigated in NMCMs expressing SRSF9‐WT/SRSF9‐△RRM1/SRSF9‐△RRM2 or control. Agarose gel electrophoresis with the percent spliced‐in (PSI) primers shown in Figure S13D (Supporting Information). O) Binding of NFATc4 pre‐mRNAs with SRSF9 was examined by RNA‐immunoprecipitation assay in NMCMs exogenously expressed Flag‐SRSF9‐WT/Falg‐SRSF9‐△RRM1/Flag‐SRSF9‐△RRM2 or control vector. The presence of NFATc4 pre‐mRNA bound to Flag‐SRSF9 was confirmed using the NFATc4‐WT primer, as illustrated in Figure S13E (Supporting Information) (n = 4). P) qRT‐PCR assay was used to detect mRNA expression of NFATc4‐L after transfection of different truncations of SRSF9 (n = 4). Q) Relative protein levels of NFATc4 in NMCMs after transfection with Flag‐tagged SRSF9 and its truncated counterpart (n = 5).

SRSF9, an RNA‐binding protein, is also involved in RNA splicing. To uncover the mechanism underlying SRSF9‐mediated cardiac hypertrophy, we predicted the genes that may bind to SRSF9 based on three databases: ENCORI, catRAPID, and KnockTF2.0. As shown in **Figure** **6**A, the intersection of these databases yielded genes known to be involved in cardiac hypertrophy, including EZH2, NFATc4, GATA4, and JARID2. To validate whether these genes are targets of SRSF9, we measured their mRNA expression in Ang II‐treated NMCMs after the knockdown of SRSF9. The results showed that Ang II produced the most remarkable elevation in NFATc4 mRNA expression, which was significantly reduced by the knockdown of SRSF9 (Figure 6B). By contrast, overexpression of SRSF9 exhibited a higher mRNA level of NFATc4 than the Adv‐Null‐treated group (Figure 6C). Western blot results showed that knockdown of SRSF9 abrogated Ang II‐induced increase in NFATc4 protein levels, while overexpression of SRSF9 increased the expression of NFATc4 (Figure 6D,E). Consistently, TAC‐induced upregulation of NFATc4 mRNA and protein levels in mouse hearts was attenuated by silencing of SRSF9 (Figure S13A,B, Supporting Information), whereas overexpression of SRSF9 in cardiac tissues resulted in a significant upregulation of NFATc4 expression (Figure S13C, Supporting Information). NFATc4 has been shown to promote cardiac hypertrophy by translocating into the cell nucleus to facilitate the transcription of fetal genes.^[^ ^22^, ^23^, ^24^ ^]^ Therefore, we examined the distribution of NFATC4 in the nucleus and cytoplasm after manipulating SRSF9. As anticipated, Ang II led to a robust nuclear accumulation of NFATc4, which was alleviated by the knockdown of SRSF9 (Figure 6F). By contrast, SRSF9 overexpression caused an increase of NFATc4 in the nuclear fraction (Figure 6G).

Given that SRSF9 is a splicing factor, we reasoned that SRSF9 may regulate the alternative splicing of NFATc4. To verify this hypothesis, we employed agarose gel electrophoresis to examine the percent spliced‐in (PSI) of two NFATc4 transcript variants (Figure S13D, Supporting Information) identified previously.^[^ ^25^ ^]^ The results showed that the PSI of a long variant transcript of NFATc4 (NFATc4‐L) was decreased by knockdown of SRSF9, suggesting SRSF9 may promote splicing of NFATc4 in a type of intron retention (Figure 6H; Figure S13D, Supporting Information). According to the RBPmap database, we predicted the potential SRSF9 binding sites (AGGAGAA and GGATGGA sequence) at intron 9 between exon 9 and 10 of NFATc4, which was marked in red font (Figure 6I). This prompted us to verify whether SRSF9 regulates NFATc4 splicing via direct binding to its pre‐mRNA. To this end, we constructed a WT minigene reporter plasmid (NFATc4‐WT) containing a genomic DNA fragment of NFATc4 exons 9–10 with intron 9 and a mutant minigene reporter plasmid with a mutation at the binding site of NFATc4 (NFATc4‐Mut) (Figure 6J). Compared with the transfected exogenic NFATc4‐WT group, mutation of the SRSF9 binding site resulted in reduced enrichment of intron retention event of NFATc4 stimulated by SRSF9 in NMCMs, suggesting that SRSF9 regulates NFATC4 splicing in a sequence‐specific manner (Figure 6J). Moreover, the RIP‐qPCR assay demonstrated that exogenous NFATc4‐WT can be bound by SRSF9, while exogenous NFATc4‐Mut lost this ability (Figure 6K).

To further examine the impact of SRSF9 on the mRNA level of NFATc4 transcript variants, we designed two pairs of primers specifically to amplify NFATc4‐L or a shorter transcript variant of NFATc4 (NFATc4‐S) respectively. qRT‐PCR results showed that the mRNA expression level of NFATc4‐L is much higher than NFATc4 pre‐mRNA and NFATc4‐S in NMCMs at baseline (Figure S13F, Supporting Information). Notably, NFATc4‐L expression was significantly upregulated in hypertrophic hearts induced by TAC, which was attenuated by silencing of SRSF9 (Figure S13G, Supporting Information). Overexpression of SRSF9 increased while SRSF9 knockdown reduced the mRNA expression of NFATc4‐L (Figure 6L,M; Figure S13H, Supporting Information). Furthermore, we examined the mRNA decay rates of NFATc4‐L and NFATc4‐S and demonstrated that the stability of NFATc4‐L was much stronger than NFATc4‐S, and knockdown of SRSF9 accelerated degradation of NFATc4‐L (Figure S13I, Supporting Information). To further elucidate which domain of SRSF9 is responsible for regulating NFATc4 pre‐mRNA splicing, we constructed two Flag‐tagged truncations of SRSF9 by removing RNA recognition domains 1 and 2 (Flag‐SRSF9‐△RRM1 and Flag‐SRSF9‐△RRM2), respectively (Figure S13J,K, Supporting Information). As shown in Figure 6N, the deletion of SRSF9 RRM1 but not the RRM2 domain markedly inhibited the alternative splicing of NFATc4. Furthermore, the binding ability of SRSF9 with NFATc4 pre‐mRNA was decreased when SRSF9 was absent in its RRM1 domain (Figure 6O). Further experiments demonstrated that deletion of the RRM1 structural domain resulted in the loss of capacity of SRSF9 to regulate the expression of NFATc4‐L mRNA and NFATc4 protein (6P, 6Q). Collectively, our data indicate that SRSF9 increases NFATc4 protein expression by regulating the splicing of NFATc4 pre‐mRNA.

### 2.7 SRSF9 Mediates Mettl1‐Induced Cardiac Hypertrophy

**Figure 7:**
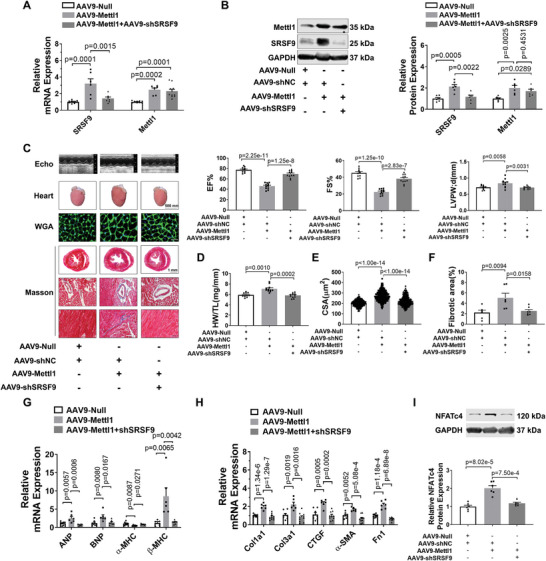
SRSF9 mediates Mettl1‐induced cardiac hypertrophy. A) qRT‐PCR analysis of Mettl1 and SRSF9 mRNA levels in heart tissues co‐injected with AAV9‐Mettl1 and AAV9‐shSRSF9 for 10 Weeks (n = 6–11). B) Western blot analysis of Mettl1 and SRSF9 protein levels in heart tissues from mice co‐infected with AAV9‐Mettl1 and AAV9‐shSRSF9 for 10 weeks (n = 6). C) Cardiac function assessed by transthoracic M‐mode echocardiography in mice co‐injected with AAV9‐Mettl1 and AAV9‐shSRSF9 for 10 weeks. Echocardiographic parameters: EF%, FS%, LVPW;d (n = 10‐11). D) Relative HW to TL in mice co‐infected with AAV9‐Mettl1 and AAV9‐shSRSF9 10 Weeks (n = 10,11). E) Quantification of cross‐sectional areas (CSA) of ventricular cardiomyocytes shown in (C). A minimum of 60 cells were measured from different microscopic fields of six samples per group. Scale bar: 20 µm. F) Masson staining of myocardium from mice co‐injected with AAV9‐Mettl1 and AAV9‐shSRSF9 for 10 weeks (n = 6–7). The relative fibrotic areas in whole heart sections are shown in (C). Scale bar: 50 µm. G) qRT‐PCR analysis on the changes of cardiac hypertrophic marker genes in heart tissues from mice co‐infected with AAV9‐Mettl1 and AAV9‐shSRSF9 for 10 weeks (n = 6–8). H) qRT‐PCR analysis of fibrotic marker genes in heart tissues from mice co‐infected with AAV9‐Mettl1 and AAV9‐shSRSF9 for 10 weeks (n = 6–8). I) Western blot analysis of NTATc4 expression in heart tissues from mice co‐infected with AAV9‐Mettl1 and AAV9‐shSRSF9 for 10 weeks (n = 6).

Next, we investigated whether Mettl1‐induced cardiac hypertrophy is mediated by SRSF9. To address this question, we performed a rescue experiment to knockdown SRSF9 in Mettl1‐overexpressing hearts by treating mice with AAV9‐shSRSF9. As anticipated, AAV9‐shSRSF9 treatment markedly inhibited the increase in SRSF9 expression induced by Mettl1 overexpression relative to the AAV‐shNC‐treated group (**Figure** **7**A,B). Strikingly, Mettl1‐induced cardiac dysfunction was largely restored by knockdown of SRSF9 (Figure 7C; Figure S14A and Table S6, Supporting Information). Knockdown of SRSF9 prevented the increase in HW/TL in Mettl1‐overexpressing hearts (Figure 7D). Furthermore, cardiac hypertrophy, as displayed by the increased CSA and hypertrophic gene expression caused by overexpression of Mettl1, was attenuated by silencing of SRSF9 (Figure 7E,G). In addition, the knockdown of SRSF9 reduced cardiac fibrosis induced by Mettl1 (Figure 7F,H). NFATc4 protein levels were significantly elevated in hypertrophic hearts and cardiomyocytes, while they were abrogated by knockdown of Mettl1 (Figure S15A–D, Supporting Information). Furthermore, the knockdown of Mettl1 inhibited the PSI of NFATc4‐L and the stability of NFATc4 mRNA, while enforced expression of Mettl1 had the opposite results in NMCMs (Figure S15E–H, Supporting Information). To corroborate the in vivo results, a gain‐and‐loss‐of‐function study was employed by manipulating Mettl1 and SRSF9 expression in NMCMs. Mettl1 caused marked increases in cardiomyocyte size and fetal gene expression, which were restored by knockdown of SRSF9 (Figure S16A–C and S14B, Supporting Information). Also, a pronounced increase in NFATc4 protein expression by Mettl1 overexpression was reduced by silencing SRSF9 (Figure 7I; Figure S16D, Supporting Information). Conversely, overexpression of SRSF9 canceled out the attenuated cardiomyocyte hypertrophic phenotype mediated by knockdown of Mettl1, as reflected by increases in cardiomyocyte size, fetal genes expression, and NFATc4 protein expression in NMCMs (Figure S17, Supporting Information).

## 3 Discussion

**Figure 8:**
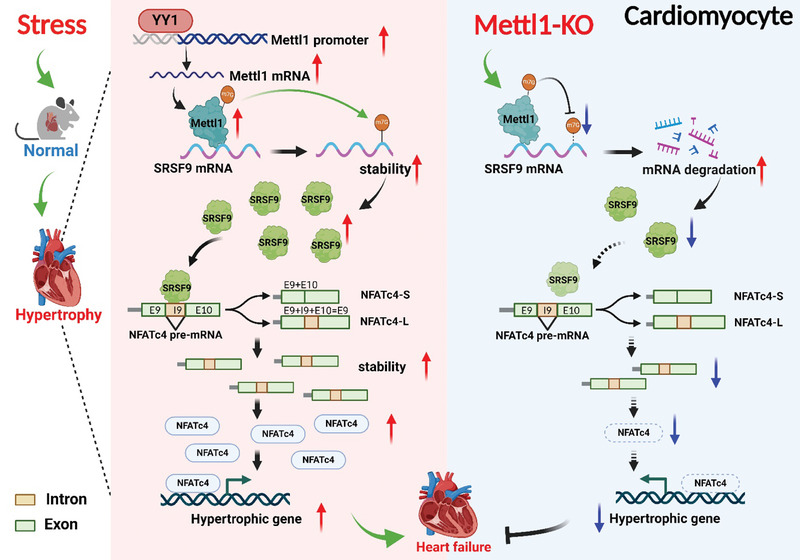
M7G methyltransferase targets the SRSF9/NFATc4 axis to regulate cardiac hypertrophy. Transcription factor YY1 is responsible for the upregulation of Mettl1 in cardiac hypertrophy. Upregulated Mettl1 methylates SRSF9 mRNA to increase its stability in an m7G‐dependent manner, which in turn promotes intron retention‐type splicing and stabilization of NFATc4, leading to cardiac hypertrophic growth. (Created with BioRender.com).

This study characterized the critical role and elucidated the molecular mechanism of Mettl1‐mediated m7G modification in cardiac hypertrophy and heart failure in the context of pressure overload (**Figure** **8**). Several new findings emerged from our study. 1) Mettl1 expression was elevated in human failing hearts and murine hypertrophic hearts, correlating with increased m7G levels in RNA. 2) YY1 acts as a transcriptional activator of Mettl1 during cardiac hypertrophy. 3) Mettl1 plays a key role in driving cardiac hypertrophy and remodeling under pressure overload conditions, as demonstrated by gain‐and‐loss‐of‐function studies. 4) Deficiency of SRSF9 exerts cardioprotective effects against TAC operation and rescues Mettl1‐induced cardiac hypertrophy. 5) Mettl1 induces m7G modification of SRSF9 mRNA, increasing the stability of SRSF9, which in turn binds to and splices NFATc4, leading to a pro‐hypertrophic phenotype. Based on these findings, the present study proposes a novel mechanistic paradigm of cardiac hypertrophy under pressure overload: TAC → Mettl1↑ → SRSF9 mRNA m7G↑ → SRSF9↑ → NFATc4↑ → hypertrophic genes↑ → cardiac hypertrophy/remodeling↑ → cardiac function↓. To the best of our knowledge, this is the first study to uncover that Mettl1 serves as a pivotal player in driving cardiac hypertrophy by regulating the SRSF9/NFATc4 axis during pressure overload.

M7G modification is dynamically regulated by Mettl1 in response to pathological stimuli.^[^ ^26^ ^]^ Upon oxidative and heat stress, an increased accumulation of m7G modifications can be observed, especially in coding sequences (CDS) and 3′UTR.^[^ ^27^ ^]^ M7G modification within mRNA is reduced in the ischemic state while significantly increased after reperfusion, which is dynamically regulated by alterations of Mettl1 expression.^[^ ^14^ ^]^ A marked increase in Mettl1 and m7G modification in total RNA has been reported in heart tissue and cardiac fibroblast after MI.^[^ ^17^ ^]^ Another study documented that mitochondrial transmembrane 11 binds to and increases Mettl1‐mediated m7G modification, thereby participating in cardiomyocyte proliferation and cardiac repair under ischemic conditions.^[^ ^16^ ^]^ By comparison, our data showed that Mettl1 is elevated in hypertrophic hearts in vivo and cardiomyocytes in vitro, in parallel with an increased m7G level. We further demonstrated that this upregulation of Mettl1 is a critical driver of cardiac hypertrophy and remodeling during pressure overload, providing a novel insight into the crucial role of Mettl1 in the context of cardiac pathology. Our results are supported by a recent report that the activation of the mTORC1 signaling pathway, a well‐established pro‐hypertrophic pathway, leads to the upregulation of Mettl1 expression in prostate cancer.^[^ ^13^ ^]^ Recently, a study screened for potential transcription factors of Mettl1 and found that SP1 and YY1 may act as transcriptional factors for Mettl1 based on the intersections of three databases.^[^ ^18^, ^20^ ^]^ However, our data demonstrated that YY1, a previously established pro‐hypertrophic transcriptional factor,^[^ ^21^ ^]^ but not SP1, transcriptionally activates Mettl1 in the context of cardiac hypertrophy.

Initially, m7G modification was primarily observed at the 5′ cap of mRNA, participating in mRNA processing.^[^ ^11^ ^]^ Mettl1‐mediated m7G modification in tRNA increases the stabilization of tRNA and enhances mRNA translation, which has been reported to be involved in the regulation of stem cell differentiation and cancer progression.^[^ ^10^, ^28^ ^]^ Besides, m7G‐methylated miRNAs such as let‐7e by Mettl1 can affect miRNA processing and maturation in tumor cells.^[^ ^29^ ^]^ Moreover, emerging evidence indicates that m7G modifications installed by Mettl1 are also observed in internal mRNA in human and mouse tissues and cell lines utilizing different high‐throughput sequencing approaches.^[^ ^11^, ^30^, ^31^ ^]^ This m7G modification in internal mRNA exhibits the ability to promote mRNA stability and translation.^[^ ^16^, ^17^, ^18^, ^32^, ^33^ ^]^ Very recent studies revealed the distribution and functional roles of m7G modification within mRNA during cardiomyocyte proliferation and cardiac fibroblast activation.^[^ ^16^, ^17^ ^]^ They showed that the blockade of Mettl1 suppressed cardiac repair and alleviated cardiac fibrosis after ischemic injury by inhibiting m7G‐methylated mRNA stability and translation. In line with these reports, our work also mapped the distribution of whole transcriptome m7G modification profiling within mRNA and identified the m7G sites in mRNAs in hypertrophic mouse hearts induced by overexpression of Mettl1 using m7G‐MeRIP‐seq and m7G AlkAniline‐Seq. Mettl1 overexpression was inhibited while knockdown of Mettl1 promoted SRSF9 mRNA decay, suggesting that Mettl1‐mediated m7G modification of SRSF9 mRNA increased SRSF9 stability and thereby SRSF9 protein expression. Further study showed that two m7G sites located in the 3′UTR of SRSF9 mRNA are crucial for the enhanced stability of SRSF9 transcript. It is well established that N6‐methyladenosine (m6A) reader proteins are responsible for recognizing m6A‐methylated mRNA, thereby regulating RNA nuclear export, splicing, degradation, translation, and stability.^[^ ^34^ ^]^ A recent study reported that Quaking proteins (QKIs) preferentially recognize internal mRNA m7G modifications. Among the QKIs, QKI7 interacts with the stress granules (SGs) core protein G3BP1 and shuttle hundreds of internal m7G‐modified mRNAs into SGs, which in turn protects them against degradation under stress conditions.^[^ ^10^ ^]^ Conversely, deletion of QKIs exhibits a global decrease in mRNA half‐life in a m7G modification‐dependent manner.^[^ ^10^ ^]^ This suggests that QKI7 may act as m7G reader protein to recognize and enhance SRSF9 mRNA stability during cardiac pressure overload, and this needs to be explored in future studies.

The serine/arginine‐rich (SR) protein family consists of 12 human SR proteins, which share common functional domains. These domains include one or two N‐terminal RNA‐binding domains (RBDs), also known as RNA recognition motifs (RRMs), that provide RNA‐binding specificity, and a C‐terminal domain rich in Arg‐Ser dipeptides (RS domain), which act as nuclear localization signals and facilitate interactions between different splicing factors.^[^ ^35^, ^36^, ^37^ ^]^ Some SR proteins have been implicated in cardiac development. For instance, cardiac‐specific deletion of SRSF1 and SRSF2 in mice leads to abnormalities in excitation‐contraction coupling and rapid onset of cardiomyopathy within four 4 weeks after birth, attributed to altered splicing of Ca^2+^/calmodulin‐dependent kinase II.^[^ ^38^, ^39^ ^]^ Similarly, knockout of SRSF10 in mice results in early embryonic lethality at E15.5, accompanied by cardiac defects and impaired intracellular Ca^2+^ handling in embryonic cardiomyocytes.^[^ ^40^ ^]^ Although SRSF9 has been shown to exhibit multiple biological functions by regulating alternative splicing, mRNA transport, stability, and translation,^[^ ^41^, ^42^ ^]^ its role in cardiac physiology and pathology has remained largely unexplored. However, a recent study has indicated that SRSF9 can activate the Wnt signaling pathway by promoting β‐catenin synthesis,^[^ ^42^ ^]^ suggesting a potential involvement of SRSF9 in cardiac remodeling. Supporting this notion, our data demonstrate that SRSF9 acts as a pro‐hypertrophy protein, and knockdown of SRSF9 rescues TAC or Mettl1‐induced cardiac hypertrophy and remodeling. In addition, our further study reveals that SRSF9 increases the production of long isoform of NFATc4 (NFATc4‐L) by regulating the splicing of NFATc4 mRNA. NFATc4‐L exhibits a higher expression level and stronger stability than NFATc4‐S. Furthermore, we demonstrate that the RRM1 domain of SRSF9 is responsible for binding to NFATc4, thereby regulating its splicing.

There are several limitations in our study that warrant further investigation. First, although we observed an elevation of Mettl1 in cardiomyocytes both in vivo and in vitro after TAC and Ang II stimulation, the expression and potential role of Mettl1 in cardiac fibroblast activation and fibrosis under non‐ischemic conditions remain to be determined. Therefore, employing cardiomyocyte and fibroblast‐specific KO mice models in the context of cardiac pressure overload in future studies would provide valuable insights into the specific roles of Mettl1 in different cell types within the heart. Second, in addition to SRSF9, we cannot exclude the possibility that other mRNAs or tRNAs may also serve as the potential targets of Mettl1 and contribute to cardiac hypertrophy. Exploring the broader landscape of Mettl1‐mediated RNA modifications in the heart and identifying additional RNA targets involved in hypertrophic signaling pathways would enhance our understanding of the molecular mechanisms underlying cardiac pathology. Lastly, while our study demonstrates that increased m7G modification of SRSF9 mRNA induced by Mettl1 enhances the stability of SRSF9, the specific reader proteins responsible for recognizing m7G modification and the underlying mechanisms governing RNA metabolism remain elusive. Further investigations are needed to identify and characterize these reader proteins and elucidate how they regulate the stability and translation of m7G‐modified mRNAs.

In conclusion, our study elucidates a previously unknown role and the molecular mechanism by which Mettl1‐mediated m7G modification contributes to cardiac hypertrophy. We demonstrate that Mettl1 enhances the stability of SRSF9 through m7G modification, leading to upregulation of NFACT4 expression and promotion of cardiac hypertrophy and heart failure progression. These findings suggest that targeting Mettl1 could represent a promising therapeutic strategy for treating heart failure. Further exploration of Mettl1 and its downstream effectors may provide valuable insights into the development of novel therapeutic interventions for cardiac hypertrophy and heart failure.

## 4 Experimental Section

### Human Heart Samples

Myocardial lysates from failing and non‐failing hearts of patients were used for gene expression analysis. Detailed information for the donors and patients has been presented in the previous study.^[^ ^43^ ^]^ The use of human cardiac tissues for the present study was approved by the Ethics Committee of the Harbin Medical University IRB2016724). The study protocols complied with the guidelines for the use of human tissues outlined in the Declaration of Helsinki.

### Animals

Neonatal C57BL/6 mice were obtained from the Laboratory Animal Center of the Second Hospital of Harbin Medical University. C57BL/6 mice weighing 20–22 g and aged 8–10 weeks were purchased from Liaoning Changsheng Biotechnology Co. Mettl1^+/−^ mice (heterozygous Mettl1 KO mice) of C57BL/6J background were purchased from Suzhou Cyagen Biosciences lnc, and sex‐matched wild‐type (WT) littermates were used as controls. The mice were housed within a dedicated pathogen‐free environment with room temperature of 23 ± 2 °C, humidity of 55 ± 5%, 12 h light/12 h dark cycle, and free access to food and sterile water. The protocols for animal experiments were approved by the Ethics Committee of Harbin Medical University (IRB3006822) and complied with the NIH Guide for the Care and Use of Laboratory Animals. The gene identification and validation of Mettl1^+/−^ mice are illustrated in Figure S4 (Supporting Information). cDNA was extracted from the mouse tail, and the primer pairs used for identifying WT and KO genotypes are presented in Table S1 (Supporting Information).

### In Vivo Gene Delivery

C57BL/6 mice were randomly assigned to receive either the adeno‐associated virus serotype 9 (AAV9) virus that carries a specific plasmid for overexpressing Mettl1(NM_01 0792) (AAV9‐cTnT‐Mettl1‐3 × Flag, AAV9‐Mettl1) or a negative control plasmid (AAV9‐cTnT‐Null, AAV9‐Null) (1.5 × 10^11^ plaque‐forming units per animal) through tail vein injection. These constructs were prepared by Shanghai Genechem Co., Ltd. AAV9 carrying a short fragment for specific knockdown of SRSF9 (AAV9‐cTnT‐shSRSF9, AAV9‐shSRSF9), a negative control scrambled RNA fragment (AAV9‐cTnT‐shNC, AAV9‐shNC) and AAV9 that specifically overexpress SRSF9 (AAV9‐cTnT‐SRSF9‐3 × Flag, AAV9‐SRSF9), or a negative control plasmid (AAV9‐cTnT‐Null‐3 × Flag) were constructed by HANbio biotechnology (Shanghai, China). The virus (1.5 × 10^11^ plaque‐forming units per animal) was administered to the mice by injecting them into the tail vein.

### Transverse Aortic Constriction (TAC) Model

Briefly, mice were anesthetized with avertin (0.2 g kg^−1^; Sigma–Aldrich Corporation, United States) via intraperitoneal injection. To access the aortic arch, the second intercostal space was bluntly incised. A 5‐0 polypropylene suture was banded against a 26 G needle around the aortic arch. Then the needle was carefully removed, and muscle and skin were sutured layer by layer to close the chest cavity by 6‐0 polypropylene suture. Sham mice underwent a similar surgical operation without aortic constriction. Ten weeks after surgery, all mice were anesthetized by intraperitoneal injection of avertin (0.2 g kg^−1^), and cardiac function was measured by echocardiography in each group, and sacrificed subsequently. A transversal slice of 3–4 mm width was obtained from the middle of the heart and rapidly preserved in 4% paraformaldehyde for histological analysis. The remaining ventricular tissue was frozen in liquid nitrogen and kept at −80 °C until use.

### Angiotensin II (Ang II) Infusion Model

Ten‐week‐old male mice (WT or Mettl1^+/−^) were treated with Ang II (2.5 mg kg^−1^ per day, Ang II, Sigma) or saline (0.9% NaCl) for 4 weeks by osmotic minipump (2002, Alzet, USA). Four weeks after the treatment, the cardiac function of the mouse was measured by echocardiography in each group, and sacrificed subsequently. A transversal slice of 3–4 mm of width was cut in the middle of the heart and rapidly fixed in 4% paraformaldehyde for histology. The rest of the ventricular tissue was frozen in liquid nitrogen and stored at −80 °C until use.

### Echocardiographic Analysis

Echocardiographic analyses were conducted to evaluate left ventricular function in mice as described previously.^[^ ^44^ ^]^ The Vevo2100 high‐resolution echocardiographic system (VisualSonics, Toronto, ON, Canada) was used with an MS400 probe and at a detection frequency of 10 MHz. Mice were anesthetized with avertin and placed on an operating table at a constant temperature of 37 °C while maintaining a heart rate of 500/min or more. Ejection fraction (EF%) and fractional shortening (FS%) were determined from M‐mode tracings. End‐diastolic LV internal dimension (LVID;d) and systolic LV internal dimension (LVID;s) were assessed at maximal and minimal diameters, respectively. The EF% was calculated as the equation (Left ventricular end‐diastolic volume – Left ventricular end‐systolic volume)/ Left ventricular end‐diastolic volume × 100%. The FS% was calculated as the equation (LVID;d – LVID;s)/LVID;d × 100%. LVPW;s is the diastolic left ventricular posterior wall thickness. Statistical analysis was performed on the average of three cardiac cycles. For the TAC model, the severity of contraction was assessed 7 days after surgery by measuring the maximal flow velocity over the ligature with an MS400 probe of 10 MHz using a continuous Doppler. Mice with a maximum flow velocity of around 3000 mm s^−1^ were included in the study.

### Tail‐Cuff Blood Pressure

Volumetric Pressure Recording (VPR) sensor technology (CODA, Kent Scientific BP Monitoring System) was used to non‐invasively measure tail blood pressure in mice. Briefly, mice were immobilized in a medium animal holder and then placed on a heating pad (37 °C). Data were collected by exposing the tail to the VPR sensor using Kent software. The procedure involved a checking phase of five cycles and ten sampling cycles with 10 s intervals in between.

### Masson's Trichrome Staining

After mouse anesthesia, whole hearts were isolated, weighed, fixed in 4% paraformaldehyde (Biosharp, Guangzhou, China), and embedded in paraffin. Mid‐transverse LV sections were cut into 4 µm thick slices using a paraffin sectioning machine (Thermo Fisher Scientific, Waltham, MA, USA). The sections were stained with a Masson's trichrome staining kit (Solarbio, Beijing, China) according to the manufacturer's protocol. The fibrosis fraction was calculated as a percentage of the whole section at 4× magnification (FV300, Olympus, Japan), and the fibrotic area was measured using ImageJ software.

### Wheat Germ Agglutinin (WGA) Staining

The heart tissue was sectioned in paraffin and sealed with 3% hydrogen peroxide after dewaxing. Next, antigen repair was performed by microwave heating method with sodium citrate solution at pH 6. After cooling to room temperature, sections were blocked with 5% BSA (Biosharp, Guangzhou, China). The stock solution of 1 mg mL^−1^ WGA (Invitrogen, CA, USA) was diluted 1:200 in PBS and used to stain the sections for 30 min at room temperature, followed by DAPI (Solarbio, Beijing, China) re‐staining for 20 min. The sections were photographed under a 40× microscope after sealing with fluorescence quenching mounting medium (#S36963, Invitrogen, CA, USA).

### Neonatal Mouse Ventricular Cardiomyocytes (NMCMs) Isolation and Culture

Neonatal mouse ventricular cardiomyocytes (NMCMs) were isolated from 1‐ to 3‐day‐old neonatal mice, as described previously.^[^ ^45^ ^]^ Briefly, the hearts were cut into small pieces in DMEM and then digested with 0.25% trypsin (Solarbio, Beijing, China) at 37 °C, and then the digested cell supernatants were transferred into a sterile tube with DMEM containing 10% FBS and 1% penicillin/streptomycin to terminate the digestion. The collected cell suspension was filtered with a 74 µm cell strainer filter, followed by centrifuge for 5 min at 1500 rpm at 4 °C. Then the pellet was resuspended in DMEM medium containing 10% FBS and 1% penicillin/streptomycin. The cardiomyocytes were cultured in a modified medium (DMEM) (Gibco, State of California, USA) with 10% FBS and 1% penicillin/streptomycin (Beyotime, Shanghai, China), and then cultured in a 37 °C incubator with 5% CO_2_. After 2 h, the cell suspension was placed in a 6‐well plate at a density of 1 × 10^6^ cells in each well. The addition of 5‐bromo‐2‐deoxyuridine (10 nm) was used to inhibit the proliferation of fibroblasts.

### Cell Transfection with Plasmids or siRNA and Treatment

Si‐Mettl1 and negative control RNA (siNC) were synthesized by Guangzhou RiboBio Co., Ltd. (Guangzhou, China). Plasmid CV702‐Puro carrying SRSF9 cDNA (NM_02 5573) with the CMV promoter and plasmids carrying deletion of SRSF9 RRM1 or RRM2 structural domains were constructed by GENECHEM (Shanghai, China). Furthermore, the SRSF9‐WT composed of the CDS and the 3′UTR regions of SRSF9 and SRSF9‐Mut plasmids depicted in Figure 4K–M and Figure S9F (Supporting Information) were constructed by GENECHEM. The minigene reporter plasmids containing NFATc4 pre‐mRNA wild‐type or mutant sequences were constructed by GENECHEM (Shanghai, China). Adv‐Mettl1(HBAD‐Adeasy‐mMettl1‐3xflag) and negative control adenovirus (Adv‐Null) were synthesized by HANbio Biotechnology (Shanghai, China). Based on the manufacturer's instructions, plasmid or siRNA was transfected with X‐tremeGENE transfection reagents (Roche, Basel, Switzerland). SiRNAs with a final concentration of 50 µm were transfected into NMCMs, and SRSF9 plasmid and Adv‐Mettl1 were transfected with a final concentration of 1 µg mL^−1^ and 2.5 × 10^7^ PFU mL^−1^, respectively. 24 h after transfection, NMCMs were incubated with Ang II with a final concentration of 1 µm for 48 h. The sequence of si‐Mettl1 was sense 5′‐CCAUGAUGAUCCAAAGGAU‐dTdT‐3′ and antisense 3′‐dTdT GGUACUACUAGGUUUCCUA‐5′. The target sequences of siSRSF9‐2, siYY1, and siSP1 were CTGCGTAAACTGGATGACA, CGACGGTTGTAATAAGAAGTT, and GUG CAAAUCAACAGAUCAU, respectively.

### Immunofluorescence

In vivo experiments, paraffin‐embedded heart sections (4 µm) were fixed with 4% paraformaldehyde and permeabilized with 0.5% Triton X‐100 for 1 h at RT. The tissue sections were repaired by microwave with sodium citrate antigen repair solution and enclosed with 1% BSA for 30 min at RT. Heart sections were incubated overnight in rabbit or mouse polyclonal primary antibody diluted with PBS in 4 °C and incubated with associated Alexa‐Fluor 594/488‐linked anti‐Rabbit/Mouse IgG antibody (Molecular Probes, Eugene, OR, USA) at room temperature for 1 h. Heart sections were stained with DAPI for 15 min. In vitro experiments, processing was identical to that of heart sections except that the sodium citrate antigen repair process was not required. Finally, the images were collected and processed by a fluorescence microscope (Olympus, Osaka, Japan).

### Luciferase Activity Assay

Luciferase reporter plasmids were constructed using the GV80 vector with Mettl1 promoters (GENECHEM, Shanghai, China). The luciferase reporter plasmid (2 µg) and empty plasmid were transfected into NMCMs cells at a density of 50−70% to determine fluorescence values 48 h after transfection according to the instructions of the Dual‐Luciferase System Kit (Promega, Madison, WI, USA).

### ChIP Assay

The NMCMs were treated with an appropriate amount of 16% formaldehyde in a serum‐free DMEM culture medium to reach a final concentration of 1%. Incubate for 10 min at RT in a chemical fume hood. Glycine solution (10×) was added to each dish containing cell culture media and formaldehyde to a final concentration of 1×. The cells were then washed twice with PBS and collected with PBS containing 1× Halt protease inhibitor cocktail. ChIP assay was performed according to the manufacturer's instructions using the same genus of anti‐YY1 (#66281‐1‐Ig, Proteintech, Wuhan, China, 10 µg per sample) or the negative control IgG. The immunoprecipitated DNA was then eluted for qRT‐PCR analysis.

### m7G mRNA meRIP‐Seq

M7G AlkAniline‐Seq was performed by CloudSeq Biotech Inc. (Shanghai, China) according to the published procedure with slight modifications. Briefly, poly(A)‐ enriched mRNA fraction was subjected to alkaline hydrolysis for fragmentation. RNA fragments were dephosphorylated with antarctic phosphatase (New England Biolabs, Inc., Massachusetts, USA), and then incubated in 1 m aniline for cleavage. RNA libraries were constructed with NEBNext Multiplex Small RNA Library Prep Set for Illumina (New England Biolabs, Inc., Massachusetts, USA) by following the manufacturer's instructions. Libraries were controlled for quality and quantified using the BioAnalyzer 2100 system (Agilent Technologies, USA). High throughput sequencing was performed on an Illumina HiSeq instrument. Raw data were generated after sequencing, image analysis, base calling, and quality filtering on The illumina HiSeq4000 sequencer. First, Q30 was used to perform quality control. After adaptor‐trimming and low‐quality reads were removed by cutadapt (v1.9.1) software, high‐quality clean reads were generated. Then these clean reads were aligned to the human reference genome (UCSC hg19) using bowtie2 software (v2.2.4) with end‐to‐end mode. Raw m7G counts and coverage counts were calculated by bedtools (v2.24) software and in‐house scripts, then m7G‐ratio (defined as count/coverage) and m7G‐fc (defined as m7G‐ratio/Input‐m7G‐ratio) were also calculated. Differentially m7G sites were calculated based on m7G‐fc. m7G sites were annotated with gene information by bedtools software. GO and KEGG Pathway analysis was performed based on the differential m7G site‐associated genes. And the m7G sites were visualized in IGV software (v2.64).

### Arraystar m7G‐mRNA Epitranscriptomic Microarray

Total RNA from each sample was quantified using the NanoDrop ND‐1000 and RNA integrity was assessed by Bioanalyzer 2100 or Mops electrophoresis. The sample preparation and microarray hybridization were performed based on Arraystar's standard protocols. Briefly, the total RNAs were immunoprecipitated with anti‐m7G antibody. The modified RNAs were eluted from the immunoprecipitated magnetic beads as the “IP”. The unmodified RNAs were recovered from the supernatant as “Sup”. The “IP” and “Sup” RNAs were labeled with Cy5 and Cy3 respectively as cRNAs in separate reactions using Arraystar RNA Labeling protocol. The cRNAs were combined and hybridized onto Arraystar Mouse mRNA Epitranscriptomic Microarray (8 × 60 K, Arraystar). After washing the slides, the arrays were scanned in two‐color channels by an Agilent Scanner G2505C. Agilent Feature Extraction software (version 11.0.1.1) was used to analyze acquired array images. Raw intensities of IP (immunoprecipitated, Cy5‐labeled) and Sup (supernatant, Cy3‐labeled) were normalized with an average of log_2_‐scaled Spike‐in RNA intensities. After Spike‐in normalization, the probe signals to have Present (P) or Marginal (M) QC flags in a certain proportion was retained for further “m7G quantity” analyses. “m7G quantity” was calculated for the m7G methylation amount based on the IP (Cy5‐labeled) normalized intensities. Differential m7G‐methylated RNAs between two comparison groups were identified by filtering with the fold change and statistical significance (p‐value) thresholds. Hierarchical Clustering was performed to show the distinguishable m7G‐methylation pattern among samples.

### Western Blotting

Western blot analysis was performed as described previously. A lysis buffer (Beyotime, Shanghai, China) containing 1% protease inhibitor and 10% phosphatase inhibitor was used to extract the total protein. The concentration of protein was measured by a bicinchoninic acid (BCA) protein kit (Beyotime, Shanghai, China). Proteins (35–50 µg) were separated by 10% SDS‐PAGE and proteins were transferred onto nitrocellulose membranes (PALL, New York, USA). Next, the membrane was incubated overnight at 4 °C with the primary antibodies as follows: anti‐Mettl1 (#ab157097, Abcam, Cambridge, UK, 1:1000), anti‐SRSF9 (#A12538, ABclonal, Wuhan, China, 1:1000), anti‐NFATc4 (#A17511, ABclonal, Wuhan, China, 1:1000), anti‐β‐Actin (#bs‐0061R, Beijing, China, 1:1000), and anti‐GAPDH (#TA‐08, Zsbio, Beijing, China, 1:1000). Next, the membranes were incubated with the fluorescence‐conjugated anti‐rabbit IgG or anti‐mouse IgG secondary antibody (1:10 000, LI‐COR Bioscience, Lincoln, USA) for 1 h in darkness. The quantification of the band intensity and the measurement of the gray value was performed by an Odyssey infrared imaging system.

### RNA Immunoprecipitation (RIP)

Immunoprecipitation of RNA‐binding proteins was performed using the Magna RIP RNA‐Binding Protein Immunoprecipitation Kit (#17‐700, Millipore, USA). Briefly, RIP was performed using m7G (#RN017M, MBL, Tokyo, Japan, 10 µg per sample) and SRSF9 (RN081PW, MBL, Tokyo, Japan, 10 µg per sample) or negative control immunoglobulin G (IgG; using Millipore, USA) of the same species according to the manufacturer's instructions. Finally, the immunoprecipitated RNA was subjected to reverse transcription to obtain cDNA for qRT‐PCR analysis.

### RNA Extraction and Quantitative Real Time‐PCR (qRT‐PCR)

qRT‐PCR analysis was performed as previously described. RNA samples from NMCMs and C57BL/6 mouse heart tissue were extracted by TRIzol (Invitrogen, Carlsbad, CA, USA) according to the manufacturer's instructions. The concentration and quality of RNA samples were detected by a NanoDrop ND‐2000 (Thermo Fisher Scientific, Waltham, MA, USA). After controlling the total RNA to 500 µg, the reverse transcription assay was performed by a Reverse Transcription kit (Toyobo, Japan) to obtain cDNA. SYBR Green (Toyobo, Japan) was used for qRT‐PCR to quantify Mettl1, SRSF9, NFATc4, GATA4, EZH2, Col1a1, Col3a1, CTGF, α‐SMA, Fn1, ANP, BNP, α‐MHC, and β‐MHC mRNA levels on a 7500 FAST Real‐Time PCR System (Applied Biosystems, Foster City, CA, USA). The calculation of the normalized RNA expression was performed by the comparative cycle threshold (Ct) method (2^−ΔΔCt^). Gene expression of each sample was normalized to 18S (Tissue) and β‐Actin (NMCMs) genes.

### Dot Blot

After extraction of total RNA from heart tissues or NMCMs using the Trizol method, RNA concentration was quantified using Nanodrop 2000. An equal volume of total RNA samples were spotted on nylon membranes and dried at RT for 5 min followed by UV‐crosslinking at 254 nm for 3 min in a CL‐1000 UV‐crosslinker. The membranes were washed with ddH_2_O for 5 min, stained with methylene blue solution (0.02% methylene), and photographed as a loading control. The nylon membranes were destained and blocked with 5% skimmed milk for 1 h, followed by incubation with mouse anti‐m7G (MBL, RN017M) and HRP‐conjugated goat anti‐mouse IgG secondary antibody according to the manufacturer's instructions.

### Statistical Analysis

Data are presented as means ± standard error of the mean (SEM). Unpaired Student's two‐tailed t‐test was used for comparisons between two groups, and one‐way analysis of variance (ANOVA) followed by the Tukey's post‐hoc correction for comparisons among multiple groups. Statistical analyses were performed using GraphPad Prism 7.0 software (GraphPad Software, San Diego, CA, USA). P < 0.05 was considered statistically significant.

## Conflict of Interest

The authors declare no conflict of interest.

## Author Contributions

S.Y., Z.S., and T.J. contributed equally to this work. W.J.D., B.F.Y., and S.T.Y. designed the study. B.F.Y. supervised the project. S.T.Y., Z.Y.S., T.T.J., Y.Q.L., Z.T.M., Z.Z.Q., C.H.W., N.L., F.W., and K.W.L. performed all experiments. M.X.L., M.H., X.C.P., Y.Q.J., Y.L., Y.Z.Z., S.K.D., J.H.J., X.H.D., C.H.H., W.H.L., and Y.Z. analyzed the data. W.J.D., S.T.Y.,and Z.Y.S. wrote the manuscript. All authors read and approved the final manuscript.

## Supporting information

Supporting Information

## Data Availability

Research data are not shared.
