# Supplementary material for: The m7G Methyltransferase Mettl1 Drives Cardiac Hypertrophy by Regulating SRSF9‐Mediated Splicing of NFATc4
Source: Adv Sci (Weinh). 2024 May 29;11(29):2308769. doi: 10.1002/advs.202308769 (PMC11304317; doi:10.1002/advs.202308769)
Supplement: Supplementary file 1 — Supporting Information [file ADVS-11-2308769-s001.docx]

**Supplementary Materials for**

**The m7G methyltransferase Mettl1 drives cardiac hypertrophy by regulating SRSF9-mediated splicing of NFATc4**

*Shuting Yu^1†^, ZhiYong Sun^1†^, Tiantian Ju^1†^, Yingqi Liu^1^, Zhongting Mei^1^, Changhao Wang^1^, Zhezhe Qu^1^, Na Li^1^, Fan Wu^1^, KuiWu Liu^1^, Meixi Lu^4^, Min Huang^1^, Xiaochen Pang^1^, Yingqiong Jia^1^, Ying Li^1^, Yaozhi Zhang^1^, Shunkang Dou^1^, Jianhao Jiang^1^, Xianhui Dong, Chuanhao Huang, Wanhong Li, Yizhang, Baofeng Yang^1,2,3*^ and Weijie Du^1,2,3*^*

***Corresponding authors:**

Prof. Weijie Du: duweijie@hrbmu.edu.cn; Prof. Baofeng Yang: [yangbf@ems.hrbmu.edu.cn](mailto:yangbf@ems.hrbmu.edu.cn); and Prof. Ye Yuan: yuany@hrbmu.edu.cn.

**This word file includes:**

Supplementary materials and methods: 1

Supplementary Figure: S1- S17

**Supplementary materials and methods**

**1. Isolation of adult mouse cardiomyocytes**

Isolation of cardiac myocytes was performed as previously described^[1]^. Hearts were rapidly cut off, cannulated, and perfused with Ca^2+^ free Tyrode solution (in mM): NaCl 137, KCl 5.4, NaH_2_PO_4_ 0.16, glucose 10, MgCl_2_ 0.5, HEPES 5.0, and NaHCO_3_ 3.0 (pH 7.4 adjusted with NaOH) for 5 min. The heart was then perfused with a solution containing 1% collagenases II (Gibco, State of California, USA) and 0.5% BSA (Biosharp, Guangzhou, China) until digestion was complete. The cardiomyocytes of the left ventricle from mouse heart were carefully separated with forceps, and then gradually exposed to Ca^2+^ (from 50 to 500 µM Ca^2+^ over 40 min) and plated in culture chambers for further studies.

**Supplementary Figure**


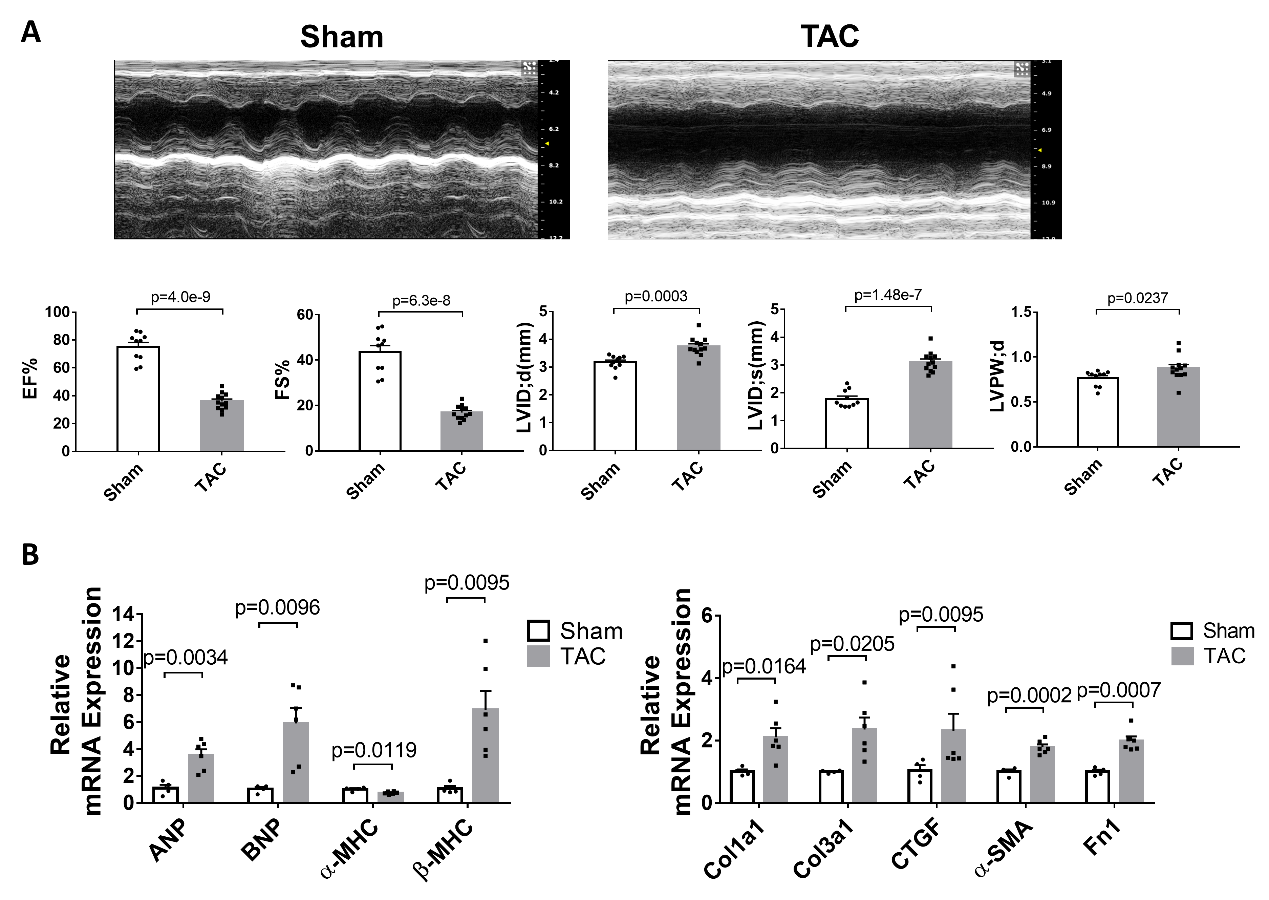


**Figure S1: Pressure overload modeling induces cardiac hypertrophy and heart failure.** (A) The transthoracic M-mode echocardiographic tracings of Sham and TAC mice for 10 weeks. Echocardiographic parameters: EF%, the ejection fraction; FS%, the fraction shortening; LVID;d or LVID;s, the internal dimension of the left ventricle (LV), diastolic or systolic; LVPW;d, the post wall thickness of LV (Sham: n=10, TAC: n=12). (B) qRT-PCR analysis for cardiac hypertrophic and fibrotic markers in sham or TAC-10 weeks heart tissue (Sham: n=4, TAC: n=6).


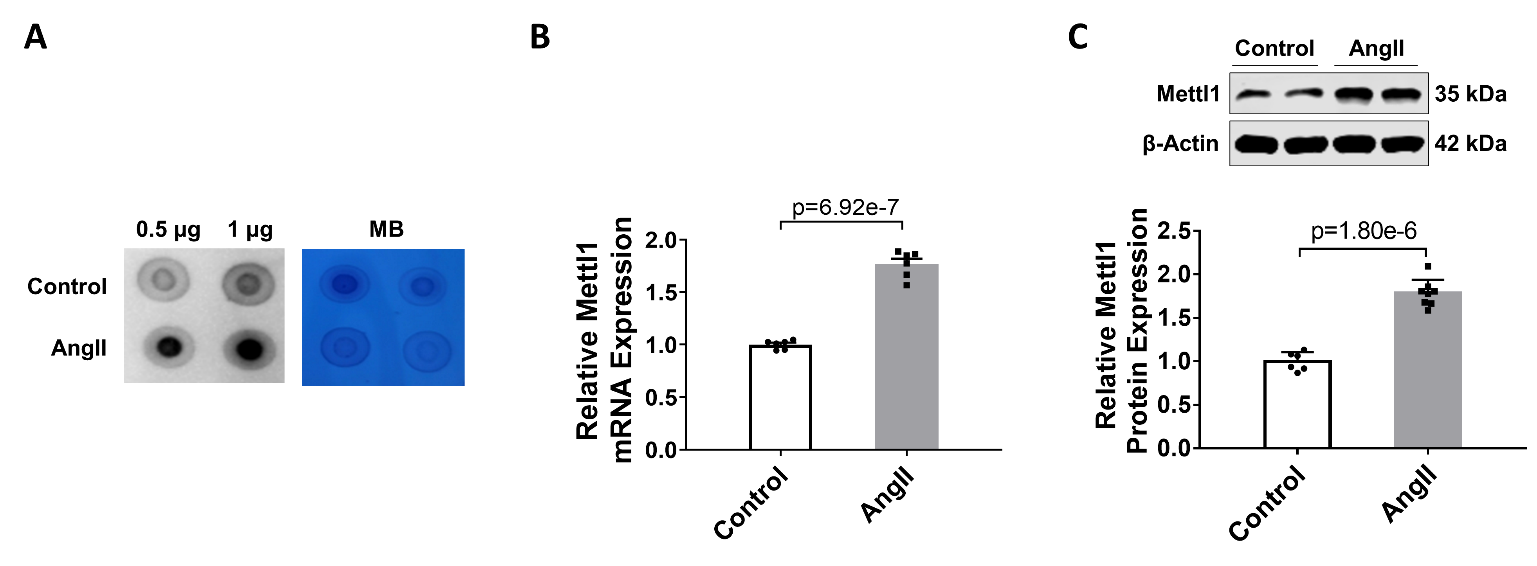


**Figure S2: Upregulation of Mettl1 in hypertrophic cardiomyocytes.** (A) Dot blot analysis of m7G modification levels in AngII-treated NMCMs for 48h, with methylene blue staining as control (n=3). (B) qRT-PCR analysis of Mettl1 mRNA levels in control or AngII-treated NMCMs for 48 h (n=6). (C) Western blot analysis of Mettl1 in control or AngII-treated NMCMs for 48 h (n=6-8).


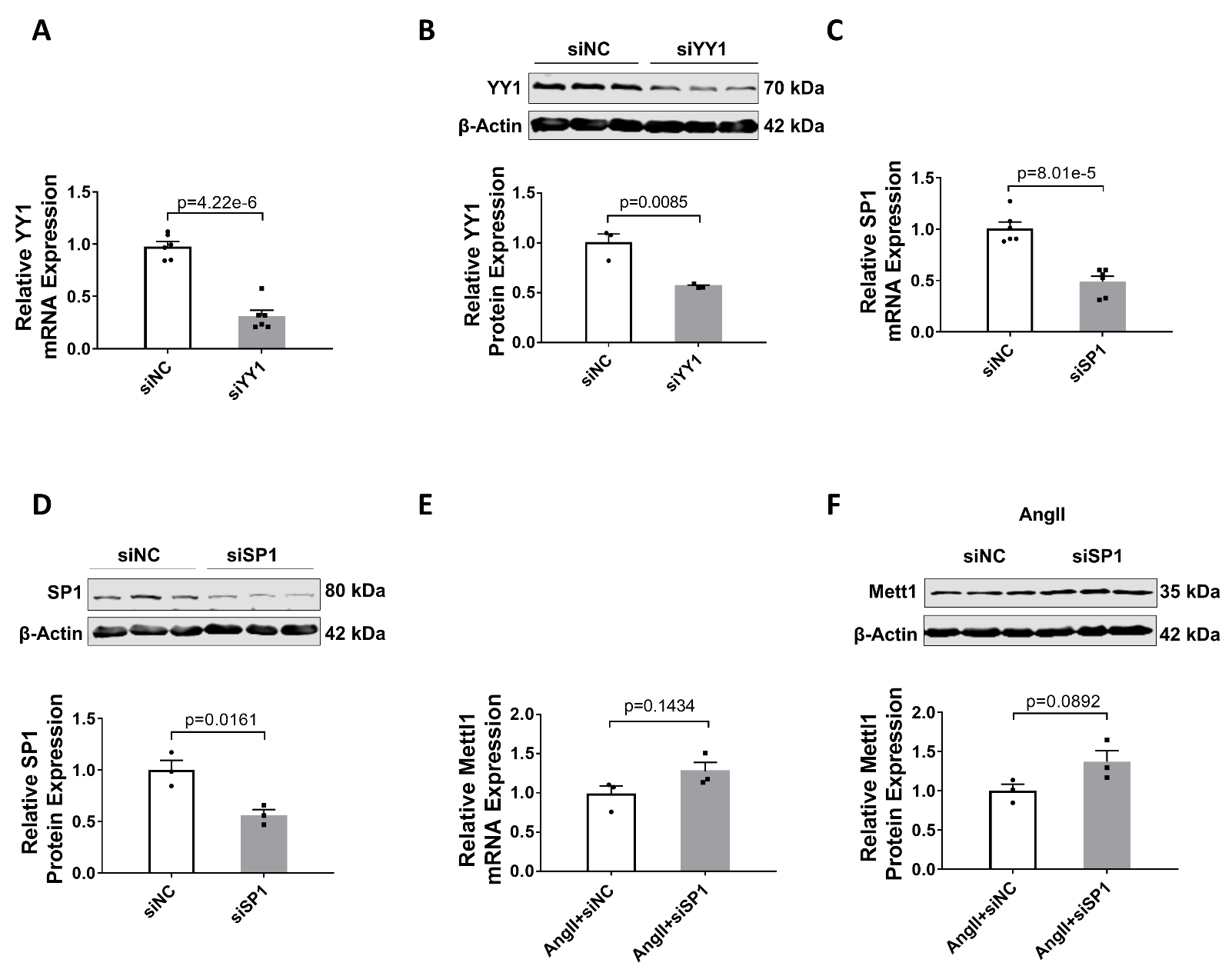


**Figure S3: Transcription factor YY1 drives upregulation of Mettl1 expression in hypertrophic cardiomyocytes.** (A) qRT-PCR analysis of transfection efficiency of siYY1 (n=6). (B) Western blot analysis of transfection efficiency of siYY1 (n=6). (C) qRT-PCR analysis of transfection efficiency of siSP1 (n=3). (D) Western blot analysis of transfection efficiency of siSP1 (n=3). (E) qRT-PCR assay was conducted to detect Mettl1 mRNA expression after silencing SP1 in AngII-treated NMCMs (n=3). (F) Western blot assay was conducted to detect Mettl1 protein expression after silencing SP1 in AngII-treated NMCMs (n=3).


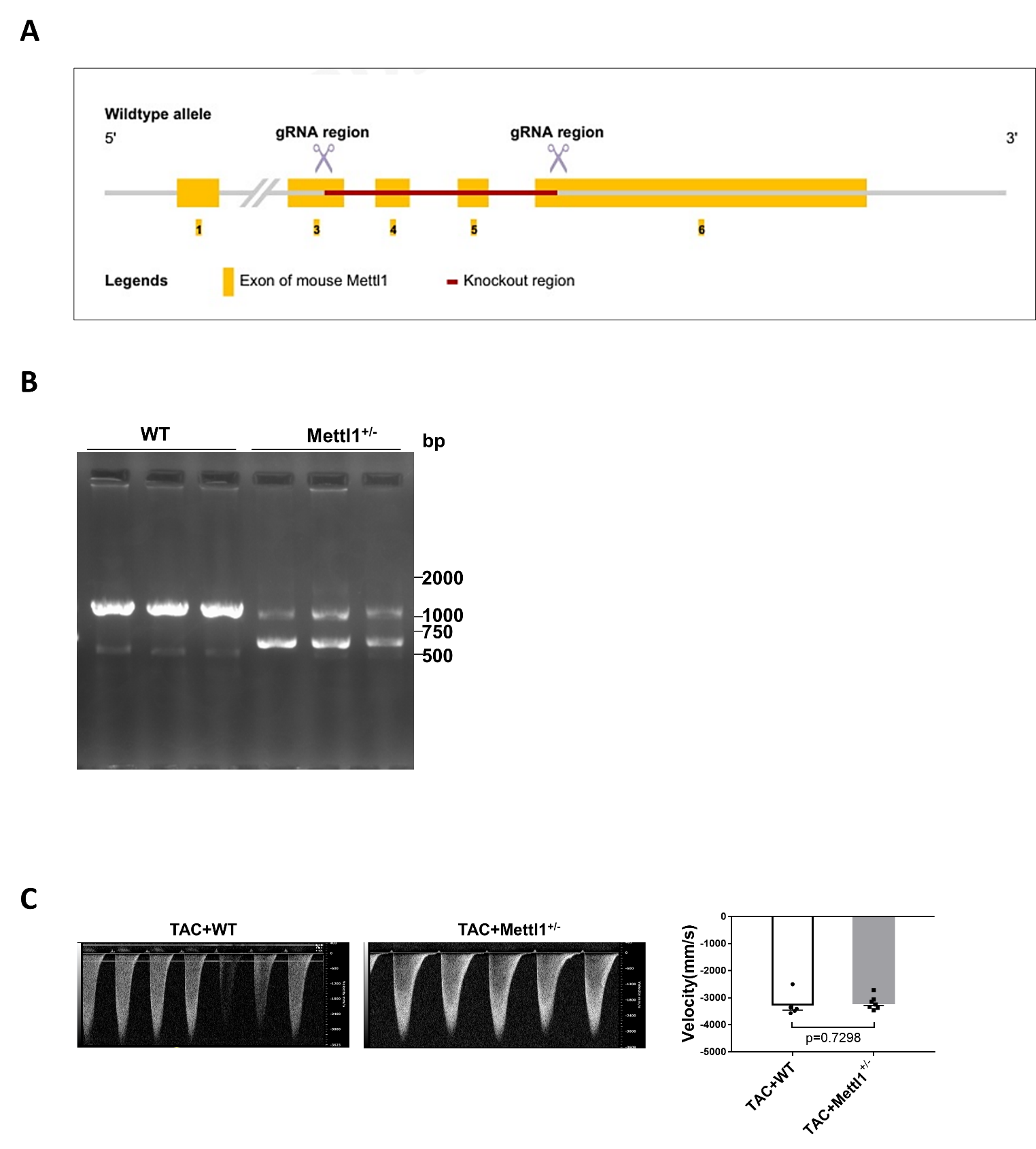


**Figure S4: Identification of Mettl1 knockout mice and echocardiographic analysis of TAC mice at 7-day points.** (A) Diagram of the constructed model of Mettl1 knockout (Mettl1^+/-^) mice. (B) Mouse genotypes were identified by PCR using the same pair of primers. The PCR product length was 1391 bp for wild-type (WT) mice and 613 bp for Mettl1^+/-^ mice. (C) Left: Representative images of echocardiography of velocity at aortic arches in TAC mice. Right: Quantitative analysis of peak velocity in TAC mice aortic arches.
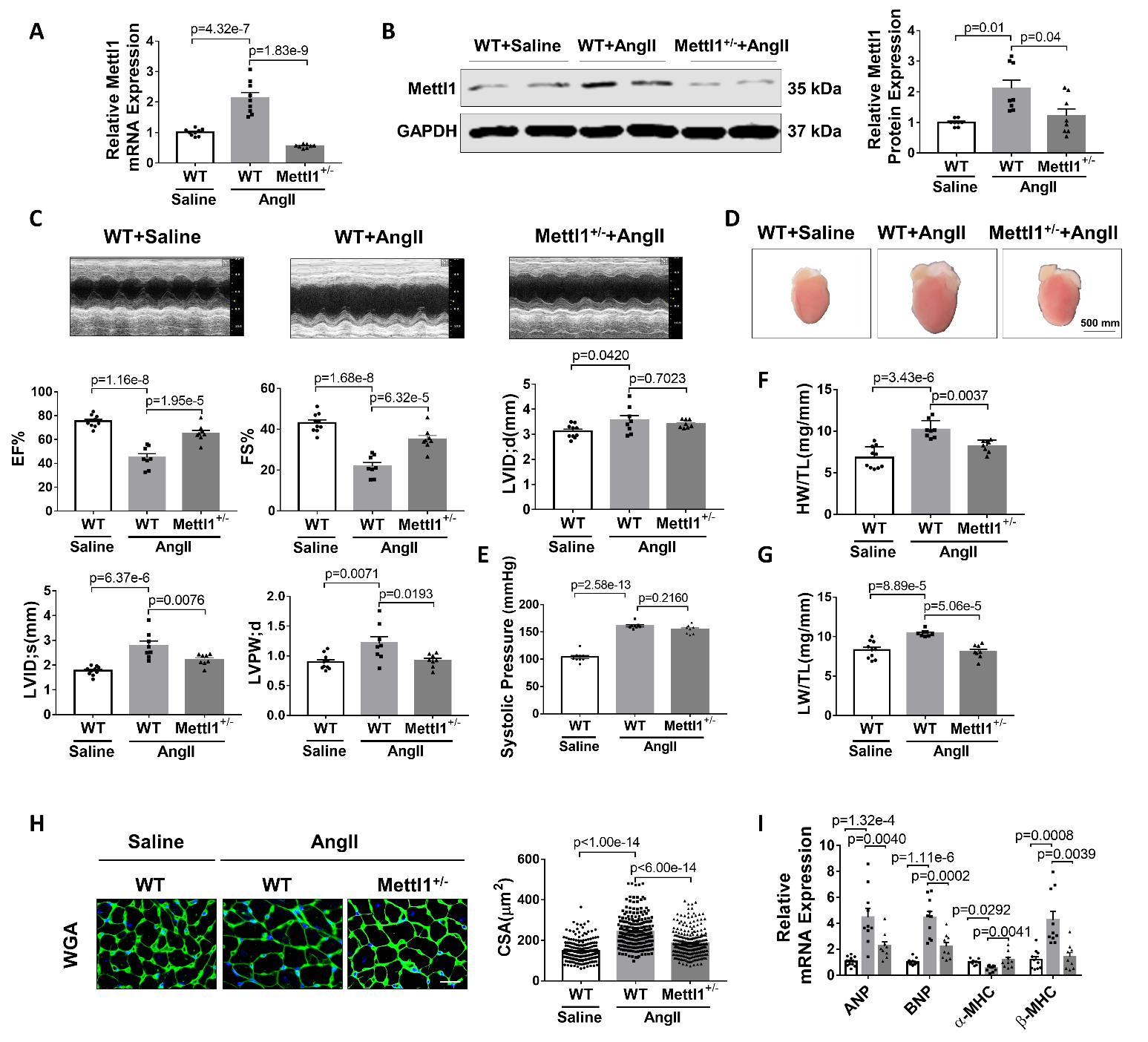


**Figure S5: Knockdown of Mettl1 ameliorates AngII-induced cardiac hypertrophy.** (A) Mettl1 mRNA expression analysis of WT and Mettl1^+/-^ heart tissue from AngII treated for 4 weeks (n=8). (B) Mettl1 protein expression analysis of WT and Mettl1^+/-^ heart tissue from AngII treated for 4 weeks (n=8). (C) The transthoracic M-mode echocardiographic tracings from WT or Mettl1^+/-^ with 4-week Saline or AngII- infusion mice. Echocardiographic parameters: EF%, FS%, LVID;d, LVID;s and LVPW;d (Saline+WT: n=10; AngII+WT: n=8; AngII+Mettl1^+/-^: n=8). (D) Representative gross morphologies of hearts from mice subjected to different treatments (Saline+WT: n=10; AngII+WT: n=8; AngII+Mettl1^+/-^: n=8). (E) Systolic blood pressure in WT or Mettl1^+/-^ with 4-week Saline or AngII-infusion mice by tail-cuff method (Saline+WT: n=10; AngII+WT: n=8; AngII+Mettl1^+/-^: n=8). (F) The relative heart weight (HW) to the tibia length (TL) of WT or Mettl1^+/-^ mice with 4-week Saline or AngII-infusion (Saline+WT: n=10; AngII+WT: n=9; AngII+Mettl1^+/-^: n=9). (G) The relative Lung weight (LW) to the tibia length (TL) of WT or Mettl1^+/-^ mice with 4-week Saline or AngII-infusion (Saline+WT: n=10; AngII+WT: n=8; AngII+Mettl1^+/-^: n=8). (H) Quantification of cross-sectional area (CSA) of ventricular cardiomyocytes. A minimum of 60 cells were measured from different visual fields of 6 samples per group. Scale bar: 20 μm. (I) qRT-PCR analysis of ANP, BNP, α-MHC and β-MHC expression in heart tissue from WT and Mettl1^+/-^ mice with Saline or AngII-infusion for 4 weeks (n=9-11).


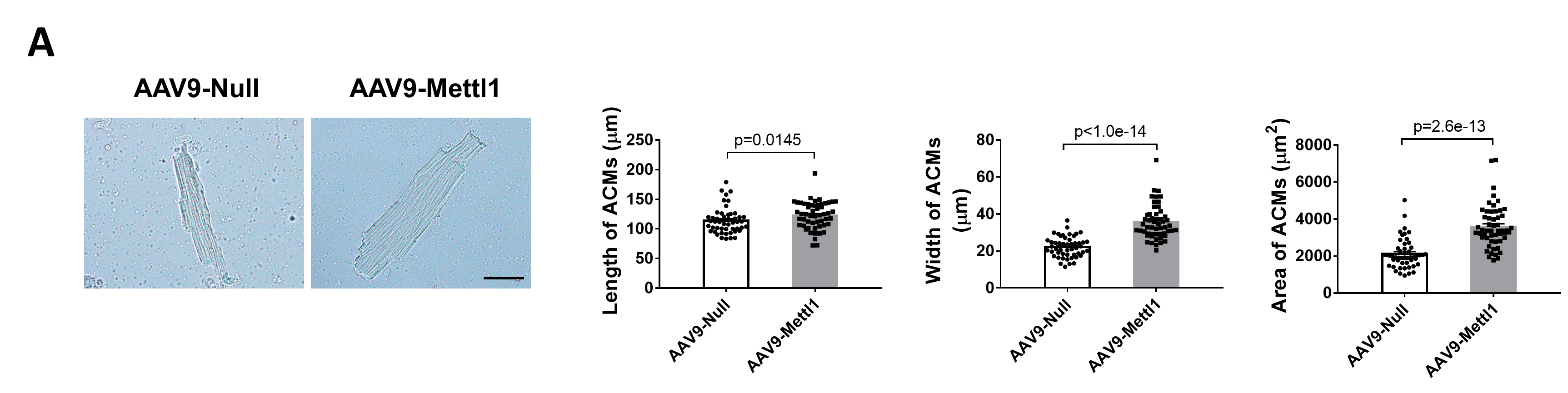


**Figure S6: Overexpression of Mettl1 promotes hypertrophy in isolated cardiomyocytes from adult mice.** (A) Representative images of cardiomyocytes from adult mice overexpressing Mettl1 and statistical plots of cardiomyocyte length, width, and area, Scale bar: 20 μm (n=50 cells from 3 mice per group).


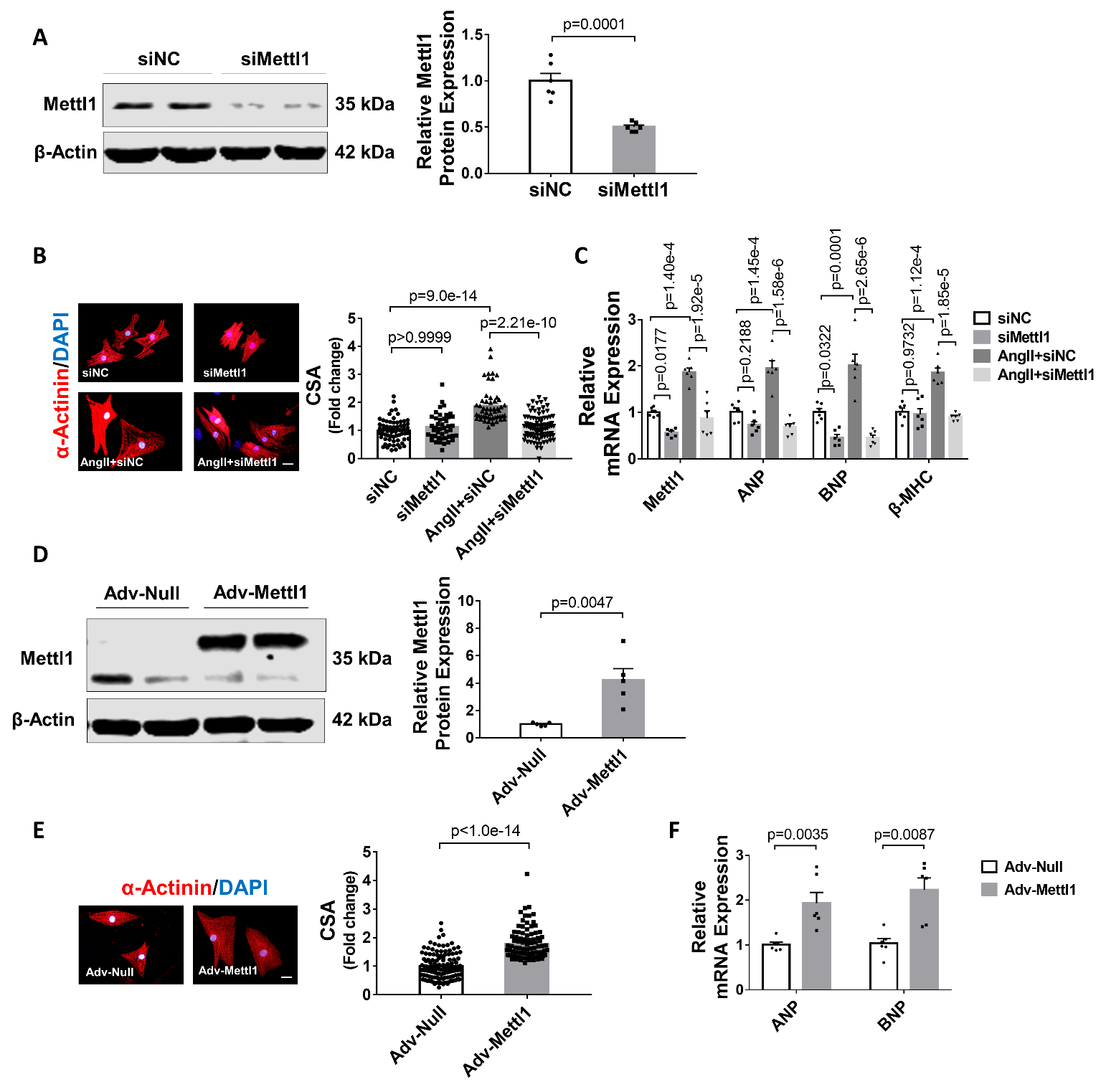


**Figure S7: Mettl1 plays a role in regulating hypertrophy of cardiomyocytes.** (A) Western blot analysis of transfection efficiency of siMettl1 (n=6). (B) Representative α-actinin-immunostaining images of NMCMs transfected with siMettl1 and treated with AngII for 48 h. A minimum of 60 cells were measured from different visual fields of three parallel experiments per group. Scale bar: 20 μm. (C) qRT-PCR analysis for cardiac hypertrophic markers in NMCMs transfected with siMettl1 and induced with AngII for 48 h (n=6). (D) Western blot analysis of transfection efficiency of Adv-Mettl1 (n=5). (E) Representative α-actinin-immunostaining images of NMCMs transfected with Adv-Mettl1. A minimum of 60 cells were measured from different visual fields of three parallel experiments per group. Scale bar: 20 μm. (F) qRT-PCR analysis for cardiac hypertrophic markers in NMCMs transfected with Adv-Mettl1 for 48 h (n=6).


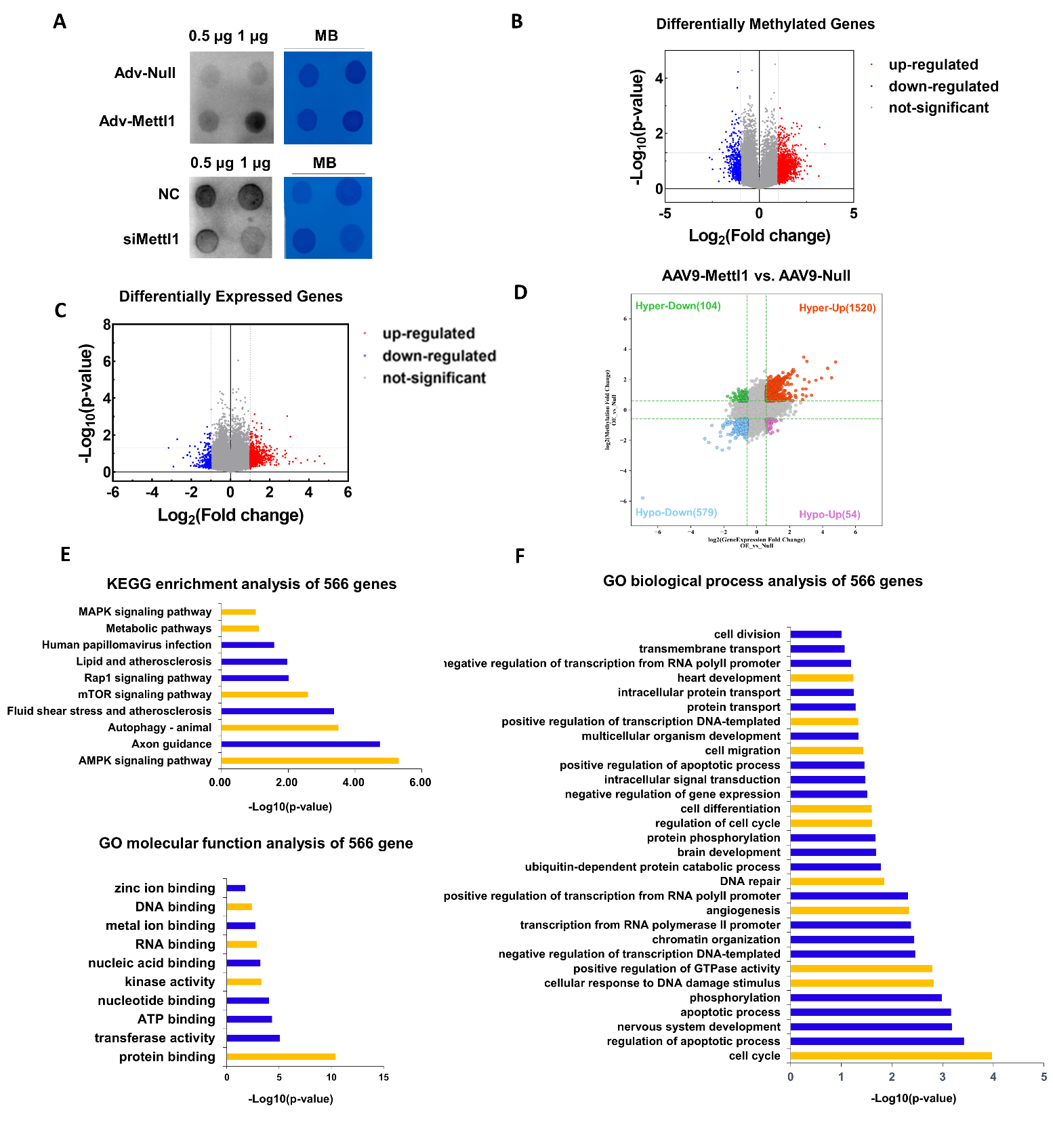


**Figure S8: Screening potential targets of Mettl1 and conducting KEGG pathway enrichment analysis and GO analysis.** (A) Top: Dot blot analysis of m7G modification levels in NMCMs transfected for Adv-Mettl1, with methylene blue staining as control (n=3). Bottom: Dot blot analysis of m7G modification levels in NMCMs transfected for siMettl1, with methylene blue staining as control (n=3). (B) A volcano plot displays differential modifications of mRNAs evaluated from m7G MeRIP-seq data. Red dots represent up-regulated genes, blue dots represent down-regulated genes, and grey dots represent genes with no significant changes. (C) Volcano plots were used to assess differentially expressed mRNAs based on mRNA-seq data from cardiac tissues that overexpress Mettl1. Red dots represent up-regulated genes, blue dots represent down-regulated genes, and grey dots represent genes with no significant changes. (D) A visualization is presented that illustrates the association between gene expression levels and changes in m7G levels in the heart of AAV9-Mettl1 mice compared to AAV9-Null mice. (E) The top 10 pathways were identified from the KEGG analysis of the 566 genes shown on the right side of Figure 5A. (F) The Gene Ontology (GO) analysis of biological processes identified the top 30 GO annotations among the 566 genes presented on the right-hand side of Figure 5A. (G) The GO analysis of molecular function identified the top 10 GO annotations among the 566 genes presented on the right-hand side of Figure 5A.


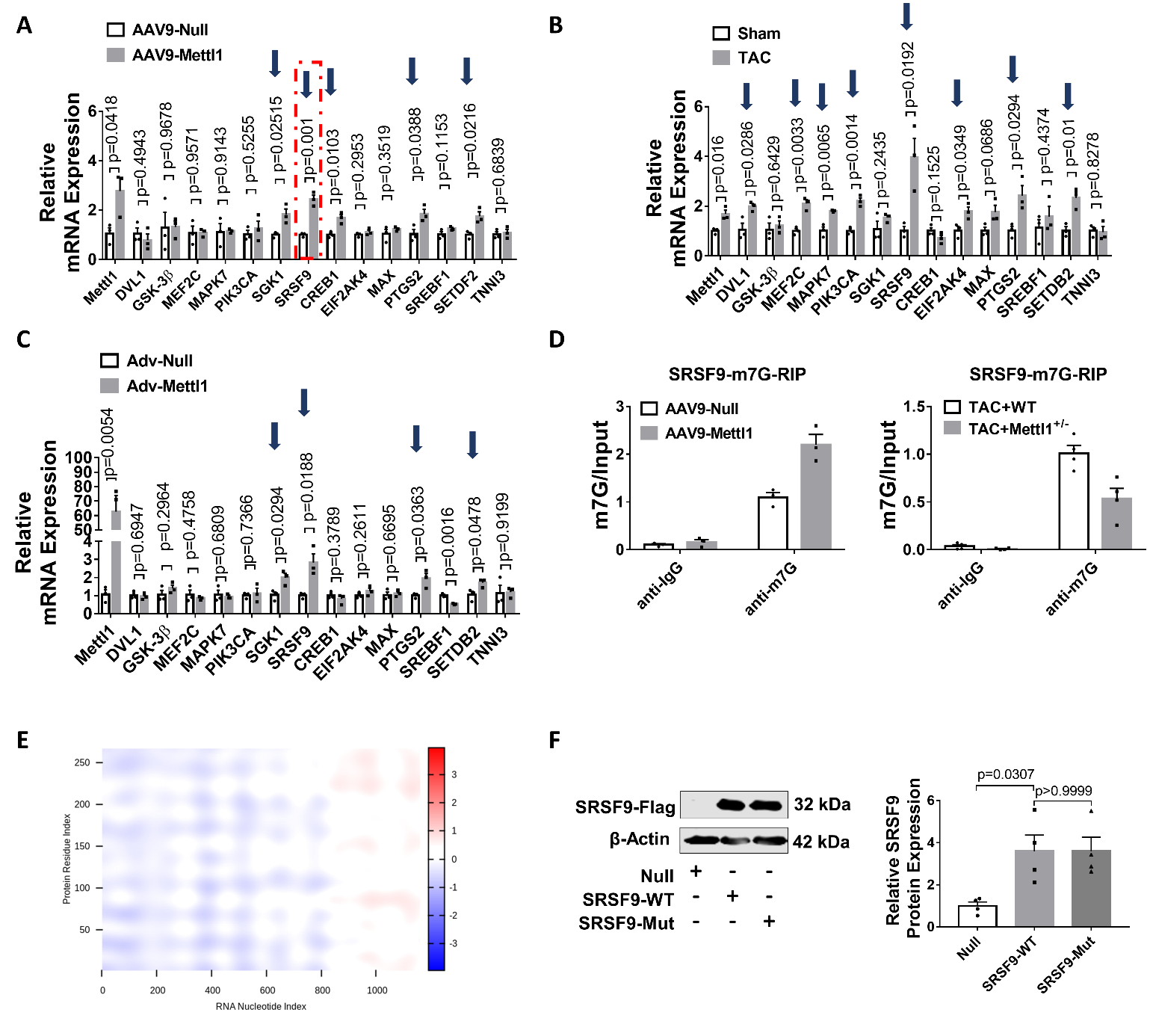


**Figure S9:** **qPCR validation of potential Mettl1 targets and detection of SRSF9 plasmid transfection efficiency.** (A) qRT-PCR analysis of m7G-MeRIP-seq differentially expressed genes in AAV9-Null and AAV9-Mettl1 mouse hearts (n=3). (B) qRT-PCR analysis of m7G-MeRIP-seq differentially expressed genes in Sham and TAC mouse hearts (n=3). (C) qRT-PCR analysis of m7G-MeRIP-seq differentially expressed genes of NMCMs transfected with Adv-Null and Adv-Mettl1 and for 48 h (n=3). (D)Left: The m7G modification effect of Mettl1 on SRSF9 was confirmed by RIP in mice injected with AAV9-Mettl1 (n=3); Right: The m7G modification effect of Mettl1 on SRSF9 was confirmed by RIP in TAC+WT and TAC+Mettl1^+/-^ mice (n=4). (E) Prediction of Mettl1's binding preference to SRSF9 mRNA by the catRAPID database. (F) Western blotting assays were performed to determine the transfection efficiency of SRSF9-WT and SRSF9-Mut containing the CDS region and the 3'UTR region in NMCMs (n=4).


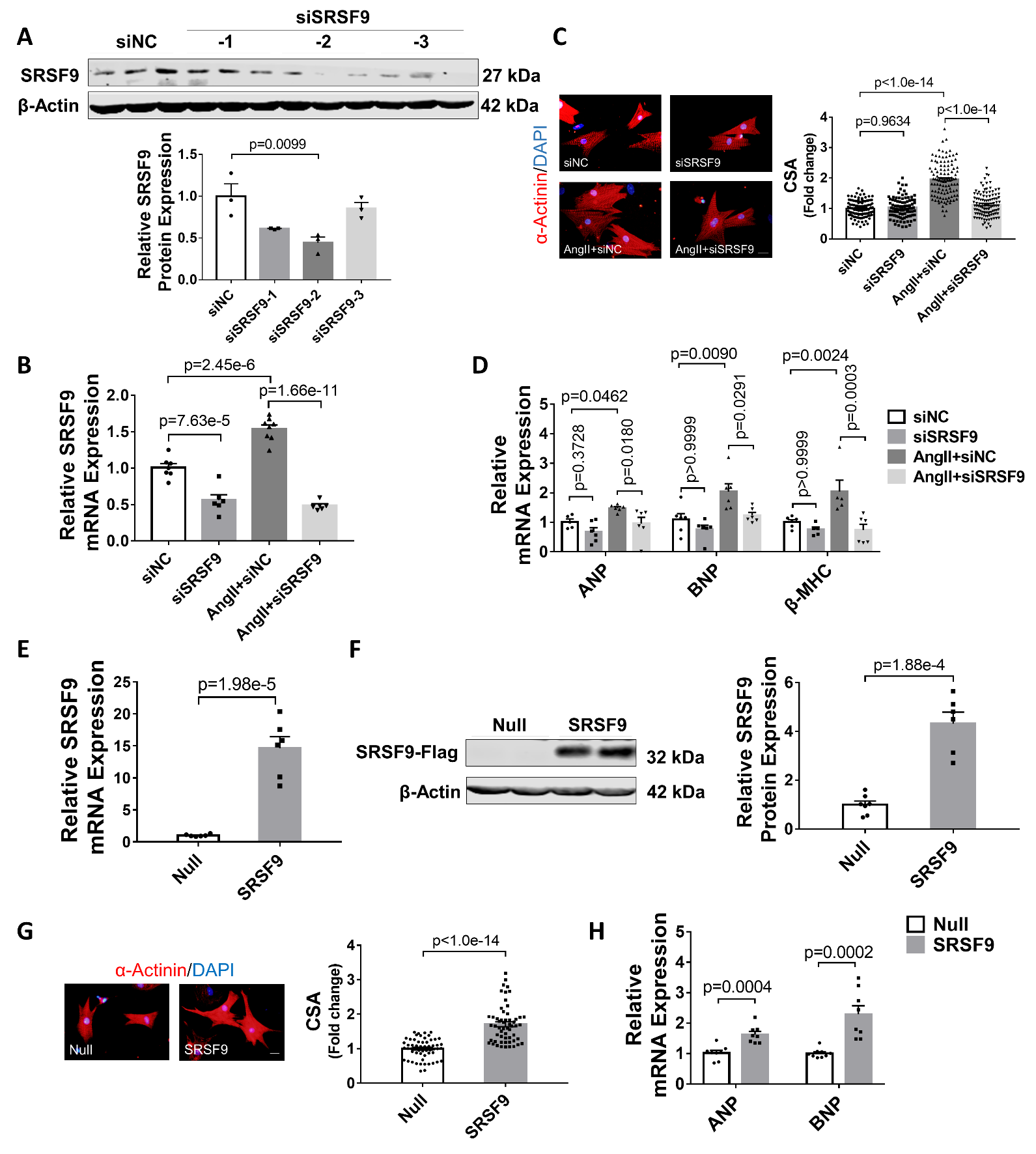


**Figure S10: SRSF9 regulates cardiomyocyte hypertrophy in vitro.** (A) Western blot analysis of transfection efficiency of siSRSF9 (n=3). (B) The transfection efficiency of siSRSF9 was determined via qPCR, with or without AngII induction (n=6-8). (C) Representative α-actinin-immunostaining images of NMCMs transfected with siSRSF9 and treated with AngII for 48 h (n≥50 cells per group from 3 parallel experiments). (D) qRT-PCR analysis for cardiac hypertrophic markers in NMCMs transfected with siSRSF9 and induced with AngII for 48 h. (E) The transfection efficiency of SRSF9-plasmid (1 μg/ml) was determined by qPCR (n=6). (F) Western blot analysis was conducted to assess the transfection efficiency of plasmids containing only the SRSF9 CDS region (n=6-7). (G) Representative α-actinin-immunostaining images of NMCMs transfected with SRSF9-plasmid (n≥50 cells per group from 3 parallel experiments). (H) qRT-PCR analysis for cardiac hypertrophic markers in NMCMs transfected with SRSF9-plasmid for 48 h (n=8-9).


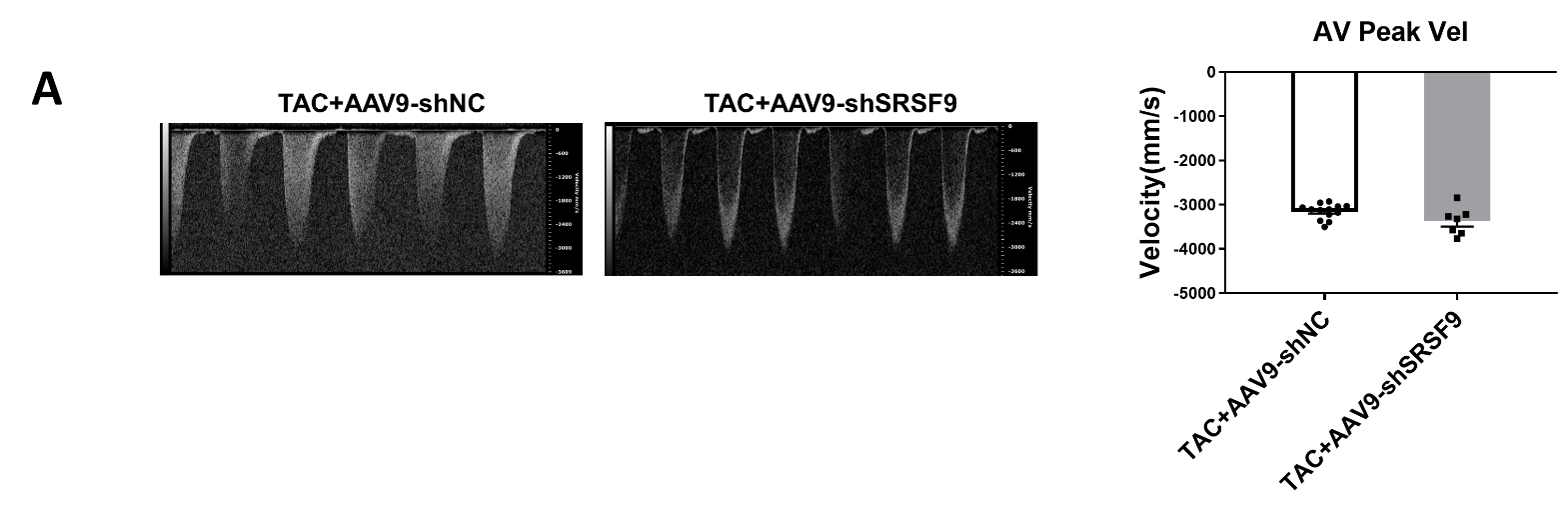


**Figure S11: Echocardiographic analysis of TAC mice at 7-day points.** (A) Left: Representative images of echocardiography of velocity at aortic arches in TAC mice. Right: Quantitative analysis of peak velocity in TAC mice aortic arches.


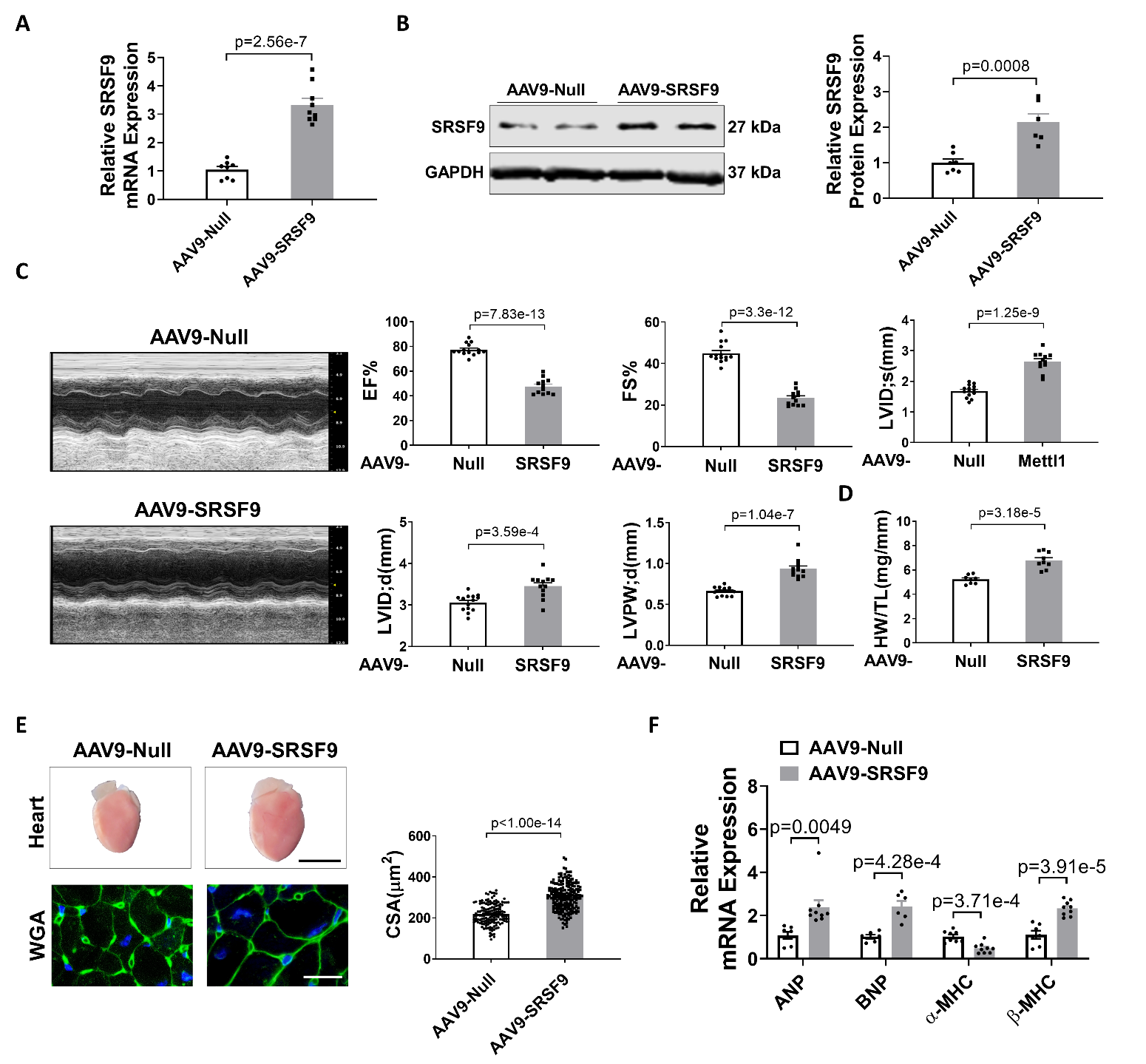


**Figure S12: SRSF9 drives cardiac hypertrophy.** (A) qRT-PCR analysis of SRSF9 mRNA expression in the myocardium from mice with AAV9-Null or AAV9-SRSF9 injection for 8 weeks (n=8-9). (B) Western blot analysis of SRSF9 protein levels in mouse hearts with AAV9-Null or AAV9-SRSF9 injection for 8 weeks (n=6-7). (C) The transthoracic M-mode echocardiographic tracings from mice injected with AAV9-Null or AAV9-SRSF9 for 8 weeks. Echocardiographic parameters: EF%, FS%, LVID;d, LVID;s, and LVPW;d (AAV9-Null: n=14; AAV9-SRSF9: n=12). (D) Relative heart weight (HW) to the tibia length (TL) of mice injected with AAV9-Null and AAV9-Mettl1 (n=8-9). (E) Quantification of cross-sectional area (CSA) of ventricular cardiomyocytes shown in F. A minimum of 100 cells were measured from different visual fields of 6 samples per group. Top: Scale bar: 500 mm/ Bottom: Scale bar:20 μm. (F) qRT-PCR analysis for the mRNA levels of cardiac hypertrophic markers in AAV9-Null or AAV9-SRSF9-treated mice (n=6-9).


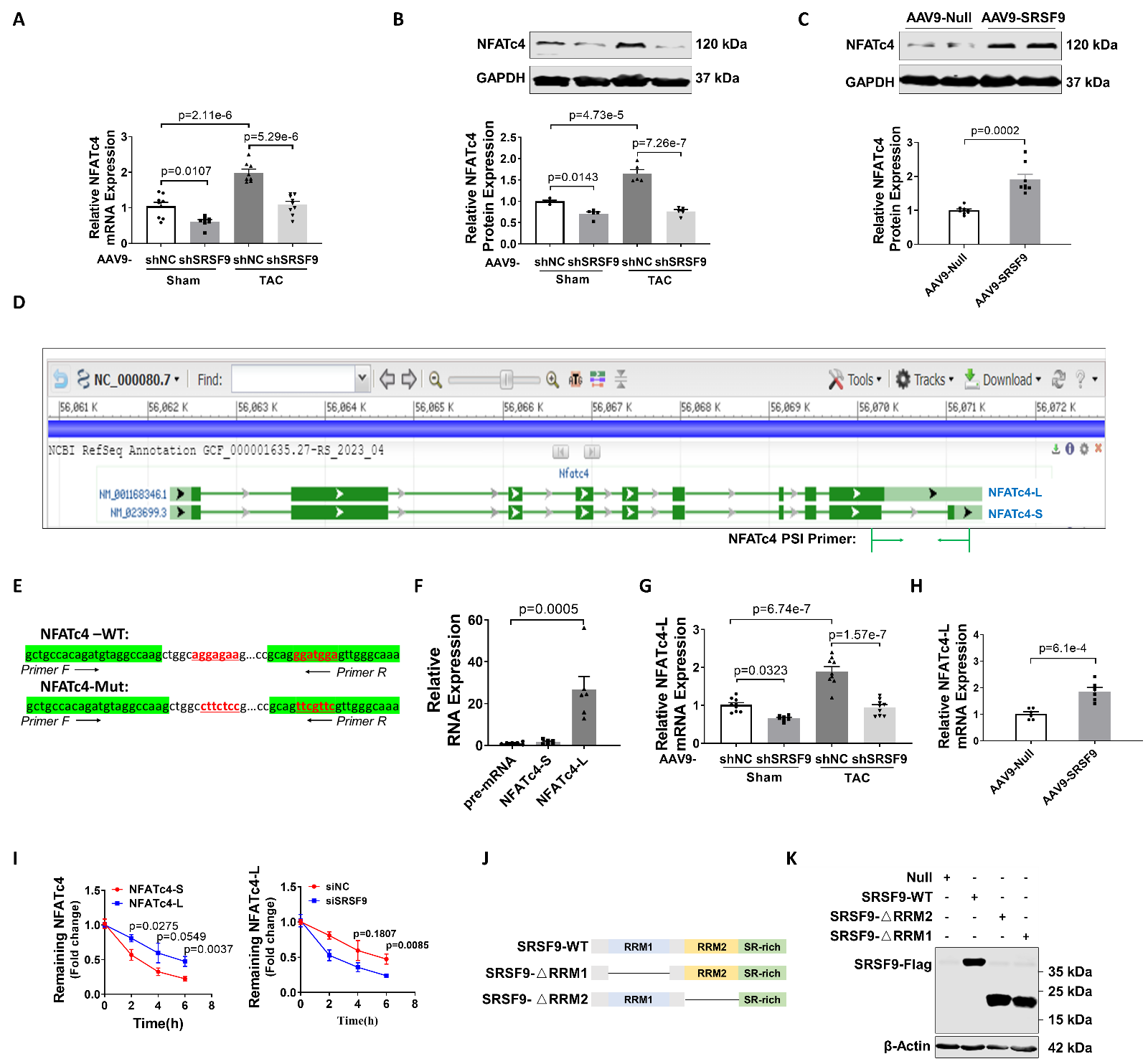


**Figure S13: SRSF9 controls the expression of NFATc4-L.** (A) qRT-PCR analysis of NFATc4 expression in heart tissue of AAV9-shNC or AAV9-shSRSF9 mice with sham and TAC for 10 weeks (n=8-9). (B) Western blot analysis of NFATc4 expression in heart tissue of AAV9-shNC or AAV9-shSRSF9 mice with sham and TAC for 10 weeks (n=5). (C) Western blot analysis of NFATc4 expression in AAV9-Null or AAV9-SRSF9-injection-8 weeks heart tissues (n=8-9). (D) Visualization of the gene for two alternative transcripts of NFATc4 (NFATc4-Shorter/NFATc4-S and NFATc4-Longer/NFATc4-L) in NCBI and a schematic diagram showing the primer design for the NFATc4 splicing efficiency experiments. (E) Primer design positions for SRSF9-RIP assays are represented by green fill colors, mutant sequences are shown in red. (F) The pre-mRNA was detected in total RNA extracted by Trizol's method by qRT-PCR (n=6). (G) qRT-PCR analysis of NFATc4-L expression in heart tissue of AAV9-shNC or AAV9-shSRSF9 mice with sham and TAC for 10 weeks (n=7-9). (H) qRT-PCR analysis of NFATc4-L expression in AAV9-Null or AAV9-SRSF9-injection-8 weeks heart tissues (n=8-9). (I) The degradation rates of NFATc4 splice variants were analyzed using qRT-PCR in NMCMs (n=7-9). (J) Schematic diagram of SRSF9 truncated variant construction. (K) The transfection efficiency of plasmids carrying full-length SRSF9 and RRM1/RRM2 structural domain deletions was determined by Western blotting (n=3).


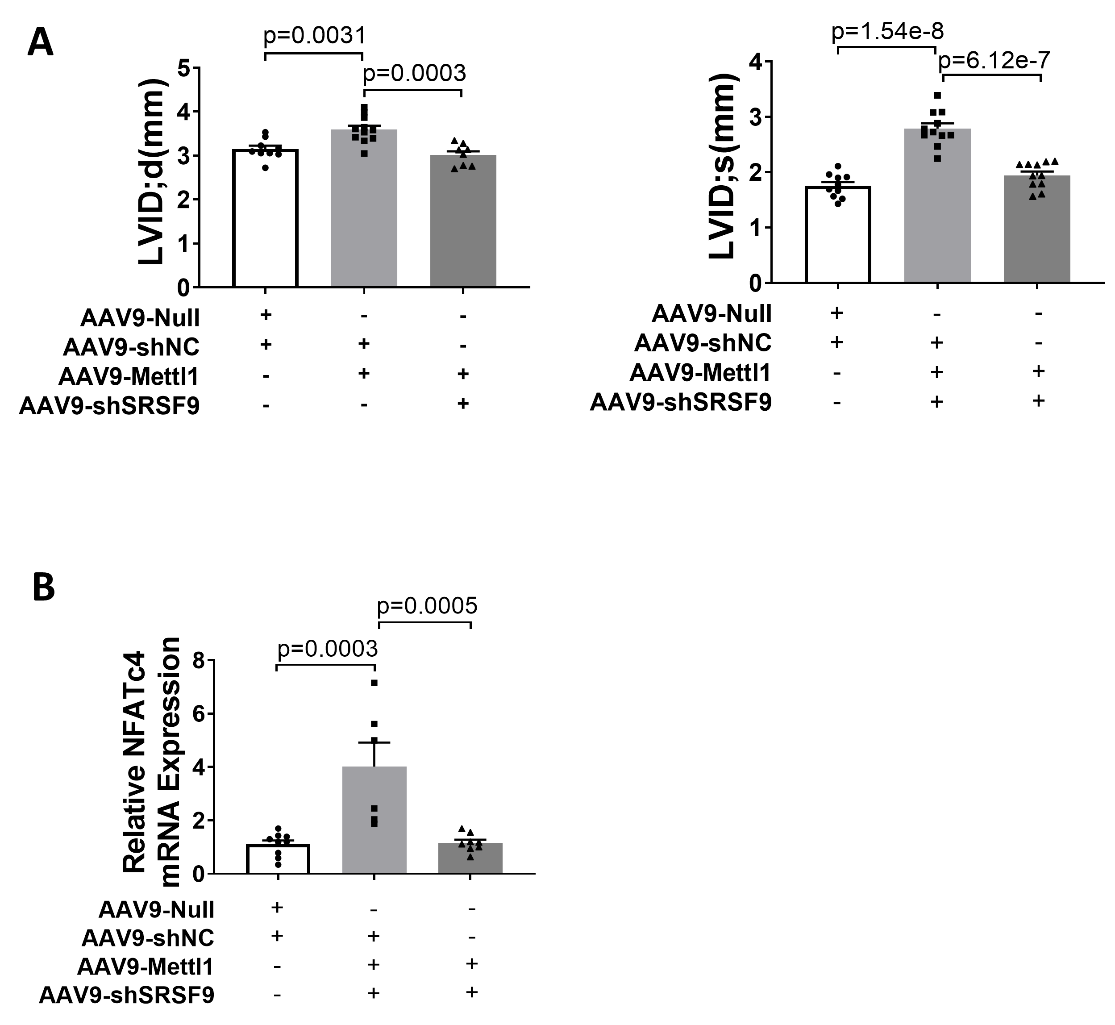


**Figure S14: Silencing of SRSF9 eliminates Mettl1's pro-cardiac dilatation effect.** (A) Cardiac function was assessed by transthoracic M-mode echocardiography in mice co-injected with AAV9-Mettl1 and AAV9-shSRSF9 for 10 weeks. Echocardiographic parameters: LVID;d and LVID;s (n=9-11). (B) qRT-PCR analysis of NTATC4 mRNA expression in heart tissues co-injected with AAV9-Mettl1 and AAV9-shSRSF9 for 10 Weeks (n=6-11).


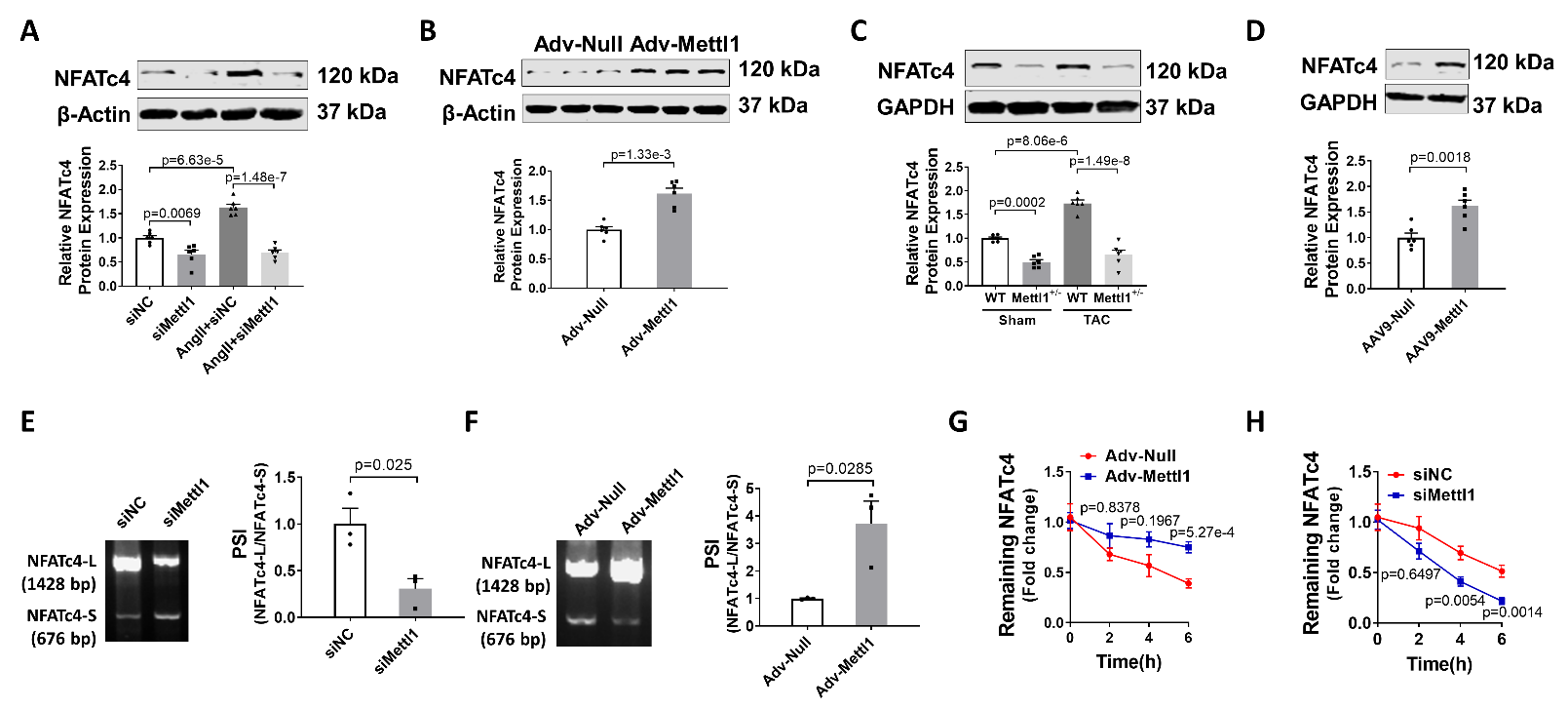


**Figure S15: Mettl1 regulates protein expression and splicing of NFATc4.** (A) After AngII treatment for 48 h, the protein expression of NFATc4 was detected in the NMCMs transfected with siMettl1 (n=6). (B) Western blot analysis of NFATc4 expression in NMCMs treated with Adv-Mettl1 for 48 hours (n=6). (C) Western blot analysis of NFATc4 expression in heart tissue from WT or Mettl1^+/-^ mice with sham and TAC for 10 weeks (n=6). (D) Western blot analysis of NFATc4 expression in AAV9-Null or AAV9-Mettl1-injection-8 weeks heart tissues (n=6). (E) Agarose gel electrophoresis was performed to determine the percent spliced-in (PSI) of NFATc4 in NMCMs silencing Mettl1(n=3). (F) Agarose gel electrophoresis was performed to determine the percent spliced-in (PSI) of NFATc4 in NMCMs overexpression of Mettl1(n=5). (G) The decay rate of NFATc4 was detected by qRT-PCR in NMCM that were treated with 5 μg/ml actinomycin D for 0, 2, 4 and 6 hours after transfection with Adv-Null or Adv-Mettl1 for 48 hours (n=6). (H) The decay rate of NFATc4 was detected by qRT-PCR in NMCM that were treated with 5 μg/ml actinomycin D for 0, 2, 4 and 6 hours after transfection with siNC or siMettl1 for 48 hours (n=6).


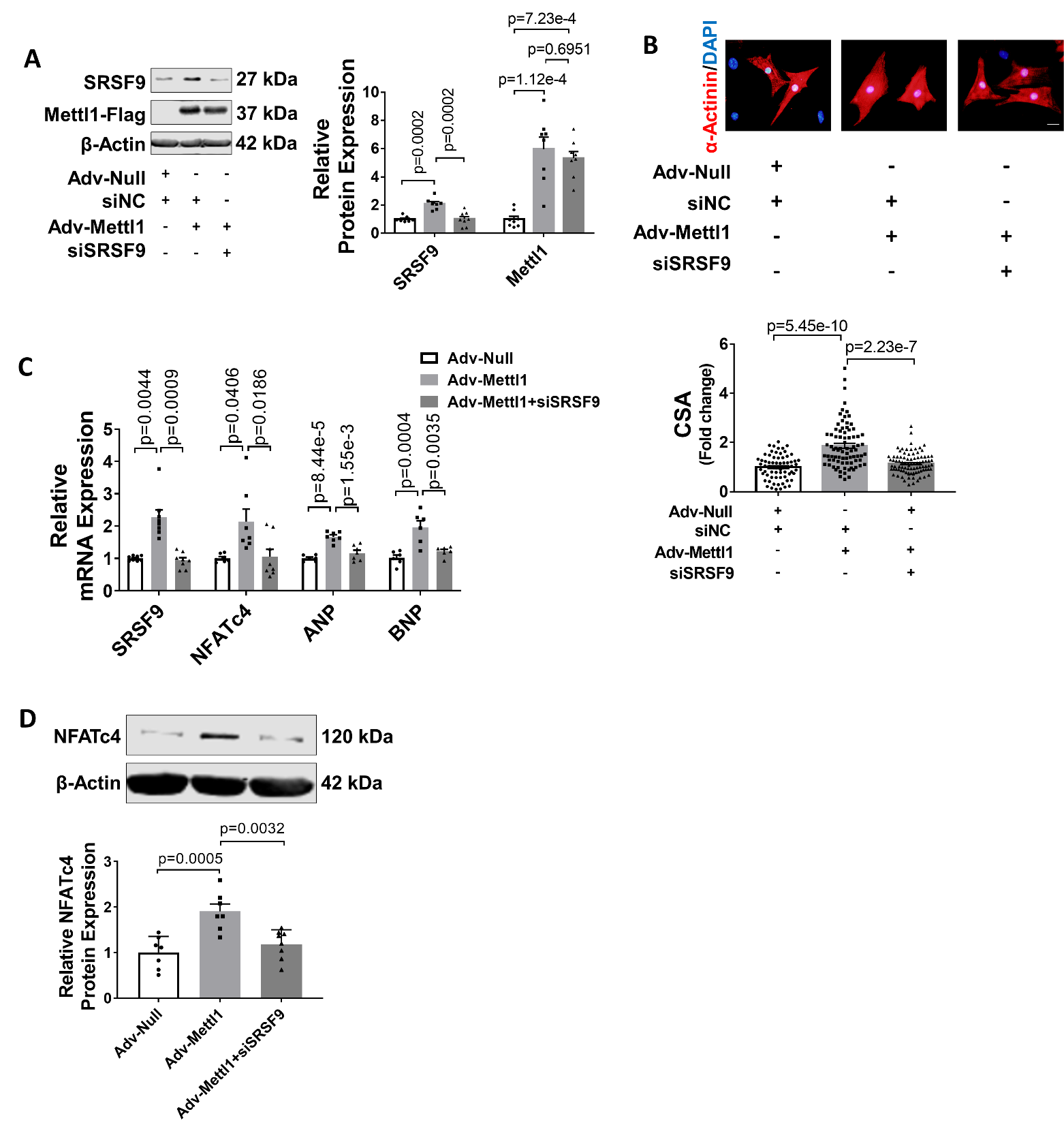


**Figure S16: Knockdown of SRSF9 restores Mettl1-induced increases in cardiomyocyte size and fetal gene expression.** (A) Western blot analysis of Mettl1 and SRSF9 expression in NMCMs co-transfected with Adv-Mettl1 and siSRSF9 for 48 hours (n=7-8). (B) Representative α-actinin-immunostaining images of NMCMs infected with Adv-Mettl1 and siSRSF9 (n≥50 cells per group from 3 parallel experiments). (C) qRT-PCR analysis for SRSF9, NFATc4 and cardiac hypertrophic markers in NMCMs of Adv-Mettl1 and siSRSF9 co-transfection (n=6-8). (D) Western blot analysis of NTATC4 expression in NMCMs co-transfected with Adv-Mettl1 and siSRSF9 for 48 hours (n=7-8).


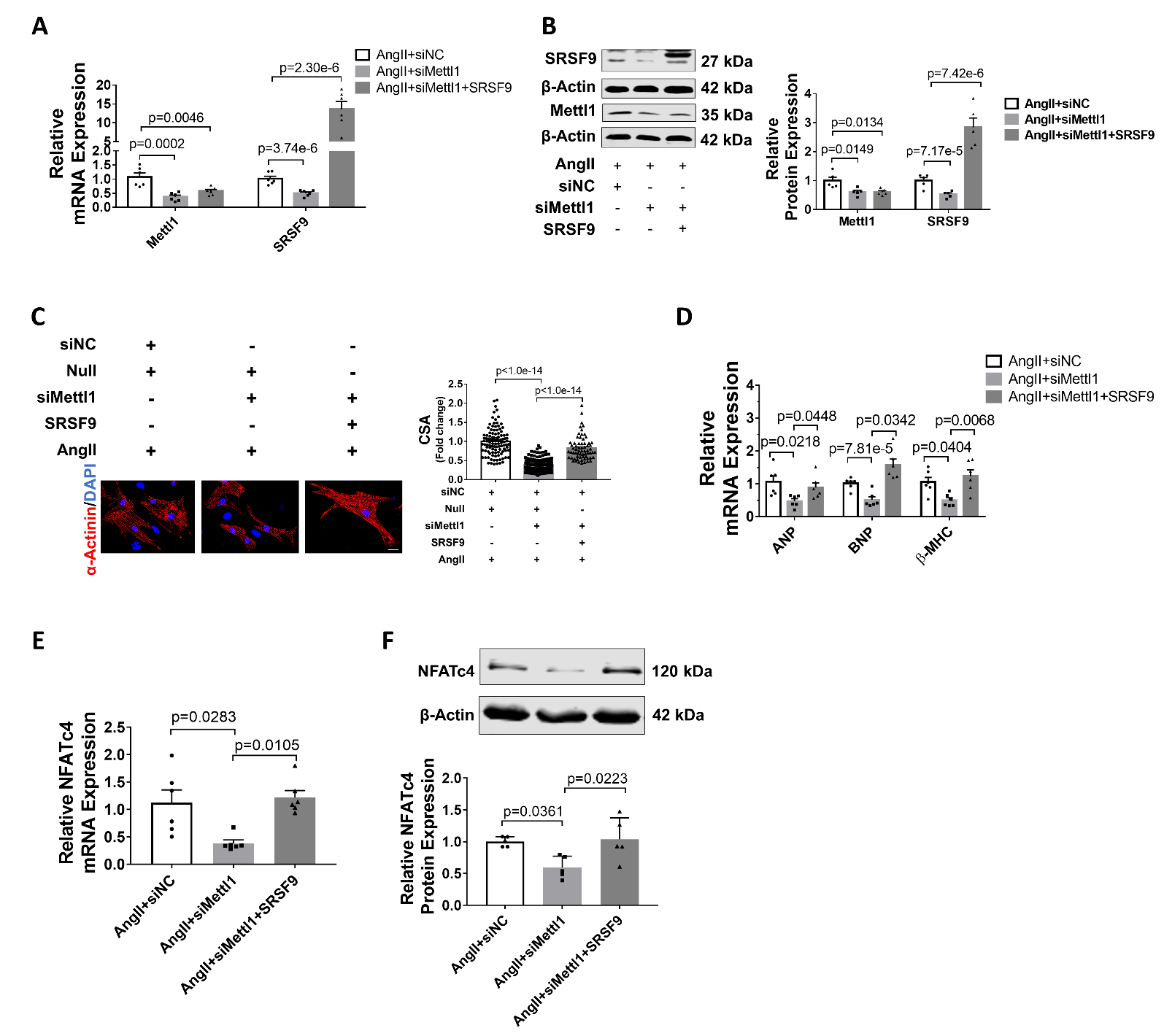


**Figure S17: Overexpression of SRSF9 canceled out the attenuated cardiomyocyte hypertrophic phenotype mediated by knockdown of Mettl1.** (A) The co-transfection efficiency of Mettl1 and SRSF9 was determined by qPCR (n=6). (B) Western blot analysis of Mettl1 and SRSF9 expression in NMCMs co-transfected with siMettl1 and SRSF9 for 48 hours (n=5). (C) Representative α-actinin-immunostaining images of NMCMs infected with siMettl1 and SRSF9 (n≥50 cells per group from 3 parallel experiments). (D) qRT-PCR analysis for cardiac hypertrophic markers in NMCMs of siMettl1 and SRSF9 co-transfection (n=6). (E) qRT-PCR analysis for NFATc4 in NMCMs of siMettl1 and SRSF9 co-transfection (n=6). (F) Western blot analysis of NTATC4 expression in NMCMs co-transfected with siMettl1 and SRSF9 for 48 hours (n=6).

1. Yu S, Sun Z, Wang X, Ju T, Wang C, Liu Y, Qu Z, Liu K, Mei Z, Li N, Lu M, Wu F, Huang M, Pang X, Jia Y, Li Y, Zhang Y, Dou S, Jiang J, Li X, Yang B, Du W. Mettl13 protects against cardiac contractile dysfunction by negatively regulating C-Cbl-mediated ubiquitination of SERCA2a in ischemic heart failure. Sci China Life Sci, 2023.

**Supplementary Materials for**

**The m7G methyltransferase Mettl1 drives cardiac hypertrophy by regulating SRSF9-mediated splicing of NFATc4**

*Shuting Yu^1†^, ZhiYong Sun^1†^, Tiantian Ju^1†^, Yingqi Liu^1^, Zhongting Mei^1^, Changhao Wang^1^, Zhezhe Qu^1^, Na Li^1^, Fan Wu^1^, KuiWu Liu^1^, Meixi Lu^4^, Min Huang^1^, Xiaochen Pang^1^, Yingqiong Jia^1^, Ying Li^1^, Yaozhi Zhang^1^, Shunkang Dou^1^, Jianhao Jiang^1^, Xianhui Dong, Chuanhao Huang, Wanhong Li, Yizhang, Baofeng Yang^1,2,3*^ and Weijie Du^1,2,3*^*

***Corresponding authors:**

Prof. Weijie Du: duweijie@hrbmu.edu.cn; Prof. Baofeng Yang: [yangbf@ems.hrbmu.edu.cn](mailto:yangbf@ems.hrbmu.edu.cn); and Prof. Ye Yuan: yuany@hrbmu.edu.cn.

**This Word file includes:**

Supplementary Table: S1-S6

**Table S1:** A table of primer sequences.

| Gene name | Forward primer (5’-3’) | Reverse primer (5’-3’) |
| --- | --- | --- |
| Mettl1-KO region | GATATGGGTATCTCTGCCCCACT | GATGGGTAAAGAACACCACAGACT |
| Mettl1 | TGTGGCTATGGTGGCTTGTT | CGACACCTTCACCCGAATCT |
| Mettl1-ChIP | CGGAACATACATGATGGTGAGGTCTG | CTCCAACTCATCTGCCTGTGCTTC |
| YY1 | GTGGTTGAAGAGCAGATCATTGG | TTGCTTAGGGTCTGAGAGGTC |
| SP1 | TCTGCAGCTACCCTGACTCC | TAATTCCCATGTTGCTGGTG |
| A-SMA | GACGCTGAAGTATCCGATAG | CCACACGAAGCTCGTTATAG |
| 18S | CCTGGATACCGCAGCTAGGA | GCGGCGCAATACGAATGCCCC |
| ANP | ATGGGCTCCTTCTCCATCAC | TCTACCGGCATCTTCTCCTC |
| BNP | GAAATGGCCCAGAGACAGCT | CCGGTCTATCTTGTGCCCAA |
| α-MHC | GTTAACCAGAGTTTGAGTGACA | CCTTCTCTGACTTCGGAGGTACT |
| β-MHC | ATGTGCCGGACCTTGGAAG | CCTCGGGTTAGCTGAGAGATCA |
| β-Actin | ACTGCCGCATCCTCTTCCT | TCAACGTCACACACTTCATGATGGA |
| Col1A1 | GCTCCTCTTAGGGGCCACT | CCACGTCTCACCATTGGGG |
| Col3A1 | GCACAGCAGTCCACCGTAGA | TCTCCAAATGGGATCTCTGG |
| CTGF | ACTGGTGCAGCCAGAAAG | GCATCTCCACCCGAGTTAC |
| FN1 | ATGTGGACCCCTCCTGATAGT | GCCCAGTGATTTCAGCAAAGG |
| NFATc4 (PSI) | CCCGCCTACCTTCAGTCTCTTTCCTTCC | CATCAACCTGTCCAGCCTGTTAGCATCAC |
| GSK-3β | ATGGCAGCAAGGTAACCACAG | TCTCGGTTCTTAAATCGCTTGTC |
| SREBF1 | GCAGCCACCATCTAGCCTG | CAGCAGTGAGTCTGCCTTGAT |
| CREB1 | AGCAGCTCATGCAACATCATC | AGTCCTTACAGGAAGACTGAACT |
| EIF2AK4 | CCCGGACATACTCCTCAGGAA | GGCTACCCACAGAGAAATGGA |
| MAX | TGCGAAGGAAAAACCATACGC | TGCACGGACTTGTTGCTCC |
| PIK3CA | CACTCGTCACCATCAAACATGA | AGGGTTGAAAAAGCCGAAGGT |
| DVL1 | ATGGCGGAGACCAAAATCATC | AACTTGGCATTGTCATCGAAGA |
| SGK1 | TGGGCTATCTGCACTCCCTAA | ATGTTGTCCCGTTATGCTCAAT |
| MEF2C | ATCCCGATGCAGACGATTCAG | AACAGCACACAATCTTTGCCT |
| MAPK7 | TCAAACACGACAATATCATCGCC | GGTTAGAGGGTTTAAGATCACGG |
| SRSF9 | AGCTGGGCTACTTTGTGCTC | TGCAGCCTAACAGGTCACTG |
| PTGS2 | TGCACTATGGTTACAAAAGCTGG | TCAGGAAGCTCCTTATTTCCCTT |
| SETDB2 | AAGGGCTGTACTATCCCATCTC | CTTTGCATGTCGTCTTTGGAAG |
| TNNI3 | AGATATGACGTGGAAGCAAAAGT | TGCAGAGATCCTCACTCTTCG |
| NFATc4 | GAGCTGGAATTTAAGCTGGTGT | GGAGGGGTATCCTCTGAGTCC |
| EZH2 | AGCACAAGTCATCCCGTTAAAG | AATTCTGTTGTAAGGGCGACC |
| GATA4 | CACCCCAATCTCGATATGTTTGA | GCACAGGTAGTGTCCCGTC |
| NFATc4-S | ACCCTGGAGGAAGTGAGTGA | CCCCAGTTAAGAGGGCAAGG |
| NFATc4-L | GCTGCGAGTGTAAGTGAGTG | AGAACTTGCTTCCGAGACCA |
| S9-WT/Mut-RIP | ACTACAAGGATGACGATGACAAGGATTACA | CAGCCTAACAGGTCACTGCTAGTATGGT |
| NFATc4-WT (RIP) | GCTGCCACAGATGTAGGCCAAG | TTTGCCCAACTCCATCCCTGC |
| NFATc4-Mut (RIP) | GCTGCCACAGATGTAGGCCAAG | TTTGCCCAACGAACGAACTGC |

**Table S2:** Gross examination and echocardiographic assessment of cardiac function in WT and Mettl1^+/-^ groups of mice at 10 W after TAC.

|  | Sham+WT  (n=9) | Mettl1^+/-^  (n=7) | TAC+WT  (N=11) | TAC+Mettl1^+/-^  (n=9) |
| --- | --- | --- | --- | --- |
| Body weight (BW, g) | 25.03 ± 0.456 | 23.47 ± 0.767 | 24.67 ± 0.570 | 25.58 ± 0.870 |
| Heart weight (HW, g) | 0.109 ± 0.002 | 0.093 ± 0.004 | 0.1815 ± 0.009^***^ | 0.1336 ± 0.003^###^ |
| Heart rate (HR, bpm) | 563.2 ± 35.260 | 524.8 ± 15.530 | 531.3 ± 32.140 | 598.4 ± 41.380 |
| EF% | 73.65 ± 1.297 | 74.71 ± 1.801 | 33.06 ± 2.990^***^ | 59.87 ± 2.978^###^ |
| FS% | 41.35 ± 1.141 | 42.45 ± 1.668 | 15.53 ± 1.496^***^ | 31.31 ± 1.981^###^ |
| LVID;s | 1.764 ± 0.035 | 1.783 ± 0.085 | 3.302 ± 0.193^**^ | 2.214 ± 0.064^#^ |
| LVID;d | 3.011 ± 0.047 | 3.095 ± 0.096 | 3.095 ± 0.096^***^ | 3.375 ± 0.137^###^ |
| LVPW;s | 1.141 ± 0.026 | 1.128 ± 0.043 | 1.105 ± 0.048 | 1.249 ± 0.032 |
| LVPW;d | 0.636 ± 0.015 | 0.617 ± 0.019 | 0.954 ± 0.039^***^ | 0.819 ± 0.025^##^ |
| IVS;s | 1.327 ± 0.035 | 1.3 ± 0.029 | 1.235 ± 0.054 | 1.426 ± 0.056^#^ |
| IVS;d | 0.749 ± 0.018 | 0.737 ± 0.025 | 0.998 ± 0.048^**^ | 1.065 ± 0.087 |
| LW/TL(mg/mm) | 7.614 ± 0.175 | 7.866 ± 0.261 | 10.93 ± 0.529^***^ | 8.595 ± 0.233^##^ |
| HW/TL(mg/mm) | 6.082 ± 0.136 | 5.255 ± 0.203 | 10.36 ± 0.504^***^ | 7.473 ± 0.176^###^ |
| HW/BW(mg/g) | 4.356 ± 0.073 | 3.977 ± 0.140 | 7.375 ± 0.375^***^ | 5.282 ± 0.235^###^ |

Values represent mean + SEM; LW, Lung weight; TL, tibia length; EF%, the ejection fraction; FS%, the fraction shortening; LVID;d or LVID;s, the internal dimension of the left ventricle (LV), diastolic or systolic; ^**^P <0.01, ^***^P <0.001 vs. Sham+WT; ^#^P <0.05, ^##^P <0.01, ^###^P <0.001 vs. TAC+WT.

**Table S3:** Gross examination and echocardiographic assessment of cardiac function in WT and Mettl1^+/-^ groups of mice at 4 W after AngII-infusion.

|  | Saline+WT  (n=10) | AngII+WT  (n=8) | AngII+Mettl1^+/-^  (n=8) |
| --- | --- | --- | --- |
| Body weight (BW, g) | 26.11 ± 1.041 | 27.11 ± 0.93 | 28.05 ± 0.274 |
| Heart weight (HW, g) | 0.113 ± 0.004 | 0.178 ± 0.007^***^ | 0.152 ± 0.005^##^ |
| Heart rate (HR, bpm) | 577.9 ± 24.62 | 583 ± 33.78 | 601.4± 39.23 |
| EF% | 75.42 ± 1.548 | 44.9 ± 3.191^***^ | 65.24 ± 2.498^###^ |
| FS% | 43.12 ± 1.44 | 21.9 ± 1.803^***^ | 35.13 ± 1.908^###^ |
| LVID;s | 1.772 ± 0.056 | 2.787 ± 0.191^***^ | 2.216 ± 0.081^##^ |
| LVID;d | 3.121 ± 0.085 | 3.556 ± 0.191^*^ | 3.413 ± 0.057 |
| LVPW;s | 1.357 ± 0.091 | 1.536 ± 0.12 | 1.29 ± 0.036 |
| LVPW;d | 0.896 ± 0.040 | 1.213 ± 0.110^*^ | 0.922 ± 0.039^#^ |
| IVD;s | 1.123 ± 0.057 | 1.365 ± 0.068^*^ | 1.234 ± 0.036 |
| IVD;d | 1.707 ± 0.061 | 1.689 ± 0.106 | 1.794 ± 0.044 |
| LW/TL(mg/mm) | 8.321 ± 0.347 | 10.43 ± 0.146^***^ | 8.108 ± 0.23^###^ |
| HW/TL(mg/mm) | 6.827 ± 0.417 | 10.2 ± 0.378^***^ | 8.21 ± 0.256^#^ |
| HW/BW(mg/g) | 4.664 ± 0.132 | 6.651 ± 0.506^***^ | 5.407 ± 0.155^#^ |

Values represent mean + SEM; ^*^P <0.05, ^***^P <0.001 vs. Saline+WT; ^#^P <0.05, ^##^P <0.01, ^###^P <0.001 vs. AngII+WT.

**Table S4:** Gross examination and echocardiographic assessment of cardiac function in in Mice 8 W After AAV9-Mettl1 and AAV9-Null injections.

|  | AAV9-Null  (n=8) | AAV9-Mettl1  (n=13) |
| --- | --- | --- |
| Body weight (BW, g) | 25.63 ± 0.527 | 25.95 ± 0.601 |
| Heart weight (HW, g) | 0.109 ± 0.002 | 0.13 ± 0.003^***^ |
| Heart rate (HR, bpm) | 562.1± 19.02 | 584.5 ± 15.21 |
| EF% | 72.09 ± 1.199 | 53.08 ± 3.017^***^ |
| FS% | 40.13 ± 0.979 | 27.07 ± 2.029^***^ |
| LVID;s | 1.902 ± 0.054 | 2.542 ± 0.091^***^ |
| LVID;d | 3.176 ± 0.064 | 3.481 ± 0.063^*^ |
| LVPW;s | 1.228 ± 0.052 | 1.094 ± 0.048 |
| LVPW;d | 0.783 ± 0.017 | 0.878 ± 0.019^**^ |
| IVS;s | 1.579 ± 0.078 | 1.609 ± 0.058 |
| IVS;d | 1.027 ± 0.08 | 1.202 ± 0.034^*^ |
| HW/TL(mg/mm) | 6.029 ± 0.105 | 77.274 ± 0.142^**^ |
| HW/BW(mg/g) | 4.272 ± 0.106 | 5.025 ± 0.159^**^ |

Values represent mean + SEM; ^*^P <0.05, ^**^P <0.01, ^***^P <0.001 vs. AAV9-Null.

**Table S5:** Gross examination and echocardiographic assessment of cardiac function in AAV9-shNC and AAV9-shSRSF9 groups of mice at 10 W after TAC.

|  | Sham+AAV9-shNC  (n=11) | Sham+AAV9-shSRSF9  (n=7) | TAC+AAV9-shNC  (n=13) | TAC+AAV9-shSRSF9  (n=12) |
| --- | --- | --- | --- | --- |
| Body weight (BW, g) | 24.36 ± 0.501 | 25.11 ± 0.577 | 25.94 ± 0.429 | 26.05 ± 0.44 |
| Heart weight (HW, g) | 0.106 ± 0.003 | 0.112 ± 0.002 | 0.164± 0.008^***^ | 0.134 ± 0.008^#^ |
| Heart rate (HR, bpm) | 555.8 ± 45.29 | 591.4 ± 53.46 | 592.2 ± 34.96 | 554.7 ± 45.24 |
| EF% | 77.14 ± 2.096 | 70.39 ± 1.988 | 40.39 ± 1.411^***^ | 63.4 ± 2.84^#^ |
| FS% | 44.88 ± 2.01 | 38.78 ± 1.664 | 19.24 ± 0.780^***^ | 33.74 ± 2.0^###^ |
| LVID;s | 1.648 ± 0.088 | 1.904 ± 0.073 | 2.873 ± 0.070^***^ | 2.036 ± 0.080^##^ |
| LVID;d | 2.976 ± 0.073 | 3.11 ± 0.084 | 3.555 ± 0.067^***^ | 3.074 ± 0.076^###^ |
| LVPW;s | 1.171 ± 0.043 | 1.091 ± 0.033 | 1.114 ± 0.027 | 1.072 ± 0.050 |
| LVPW;d | 0.668 ± 0.015 | 0.661 ± 0.013 | 0.932 ± 0.026^***^ | 0.743 ± 0.023^###^ |
| IVS;s | 1.234 ± 0.033 | 1.262 ± 0.081 | 1.195 ± 0.037 | 1.191 ± 0.036 |
| IVS;d | 0.756 ± 0.019 | 0.783 ± 0.044 | 0.994 ± 0.034^***^ | 0.838 ± 0.021^##^ |
| LW/TL(mg/mm) | 7.605 ± 0.209 | 7.977 ± 0.114 | 10.05 ± 0.517^**^ | 7.999 ± 0.210^#^ |
| HW/TL(mg/mm) | 5.763 ± 0.121 | 5.944 ± 0.090 | 9.048 ± 0.457^***^ | 7.184 ± 0.474^##^ |
| HW/BW(mg/g) | 4.357 ± 0.101 | 4.461 ± 0.111 | 6.292 ± 0.269^***^ | 5.105 ± 0.248^##^ |

Values represent mean + SEM; ^**^P <0.01, ^***^P <0.001 vs. Sham+AAV9-shNC; ^#^P <0.05, ^##^P <0.01, ^###^P <0.001 vs.TAC+AAV9-shNC.

**Table S6:** Gross examination and echocardiographic assessment of cardiac function in Mice 8 W After AAV9-Mettl1 and AAV9-shSRSF9 injections.

|  | AAV9-Null  (n=10) | AAV9-Mettl1  (n=11) | AAV9-Mettl1+AAV9-shSRSF9  (n=11) |
| --- | --- | --- | --- |
| Body weight (BW, g) | 26.66 ± 0.558 | 26.49 ± 0.73 | 25.38 ± 0.463 |
| Heart weight (HW, g) | 0.110 ± 0.003 | 0.126 ± 0.004^**^ | 0.107 ± 0.002^###^ |
| Heart rate (HR, bpm) | 559.1 ± 31.14 | 539.2 ± 22.05 | 548.6 ± 35.45 |
| EF% | 77.41 ± 1.658 | 46.12 ± 1.778^***^ | 69.36 ± 1.959^###^ |
| FS% | 45.11 ± 1.599 | 22.53 ± 1.008^***^ | 38.06 ± 1.564^###^ |
| LVID;s | 1.753 ± 0.069 | 2.786 ± 0.095^***^ | 1.942 ± 0.070^###^ |
| LVID;d | 3.146 ± 0.079 | 3.591 ± 0.092^**^ | 3.012 ± 0.088^###^ |
| LVPW;s | 1.246 ± 0.041 | 1.014 ± 0.052^**^ | 1.083 ± 0.050 |
| LVPW;d | 0.710 ± 0.022 | 0.840 ± 0.039^**^ | 0.704 ± 0.013^##^ |
| IVS;s | 1.342 ± 0.022 | 1.127 ± 0.038^***^ | 1.243 ± 0.029^#^ |
| IVS;d | 0.798 ± 0.030 | 0.812 ± 0.028 | 0.789 ± 0.027 |
| HW/TL(mg/mm) | 5.893 ± 0.137 | 7.08 ± 0.198^**^ | 5.807 ± 0.140^##^ |
| HW/BW(mg/g) | 4.14 ± 0.059 | 4.764 ± 0.099^***^ | 4.217 ± 0.0419^###^ |

Values represent mean + SEM; ^**^P <0.01, ^***^P <0.001 vs. AAV9-Null; ^#^P <0.05, ^##^P <0.01, ^###^P <0.001 vs. AAV9-Mettl1.
